# Vegetation controls on channel network complexity in coastal wetlands

**DOI:** 10.1038/s41467-023-42731-3

**Published:** 2023-11-07

**Authors:** Roeland C. van de Vijsel, Jim van Belzen, Tjeerd J. Bouma, Daphne van der Wal, Bas W. Borsje, Stijn Temmerman, Loreta Cornacchia, Olivier Gourgue, Johan van de Koppel

**Affiliations:** 1 Department of Estuarine and Delta Systems, NIOZ Royal Netherlands Institute for Sea Research, Yerseke, The Netherlands; 2 Groningen Institute for Evolutionary Life Sciences, University of Groningen, Groningen, The Netherlands; 3 Wageningen Marine Research, Wageningen University and Research, Yerseke, The Netherlands; 4 Ecosphere Research Group, University of Antwerp, Antwerp, Belgium; 5 Department of Physical Geography, Faculty of Geosciences, Utrecht University, Utrecht, The Netherlands; 6 Faculty of Geo-Information Science and Earth Observation, University of Twente, Enschede, The Netherlands; 7 Water Engineering and Management, University of Twente, Enschede, The Netherlands; 8 Operational Directorate Natural Environment, Royal Belgian Institute of Natural Sciences, Brussels, Belgium; 9 Present Address: Hydrology and Environmental Hydraulics Group, Wageningen University, Wageningen, The Netherlands; 10 Present Address: Marine and Coastal Systems, Deltares Delft, The Netherlands

**Keywords:** Complexity, Ecological modelling, Geomorphology

## Abstract

Channel networks are key to coastal wetland functioning and resilience under climate change. Vegetation affects sediment and hydrodynamics in many different ways, which calls for a coherent framework to explain how vegetation shapes channel network geometry and functioning. Here, we introduce an idealized model that shows how coastal wetland vegetation creates more complexly branching networks by increasing the ratio of channel incision versus topographic diffusion rates, thereby amplifying the channelization feedback that recursively incises finer-scale side-channels. This complexification trend qualitatively agrees with and provides an explanation for field data presented here as well as in earlier studies. Moreover, our model demonstrates that a stronger biogeomorphic feedback leads to higher and more densely vegetated marsh platforms and more extensive drainage networks. These findings may inspire future field research by raising the hypothesis that vegetation-induced self-organization enhances the storm surge buffering capacity of coastal wetlands and their resilience under sea-level rise.

## Abstract (web summary)

Channel networks are key to coastal wetland functioning. Here, the authors show how vegetation enhances network branching, and hypothesize that this may enhance the storm surge buffering capacity of wetlands and their resilience under sea-level rise.

## Introduction

**Fig. 1:**
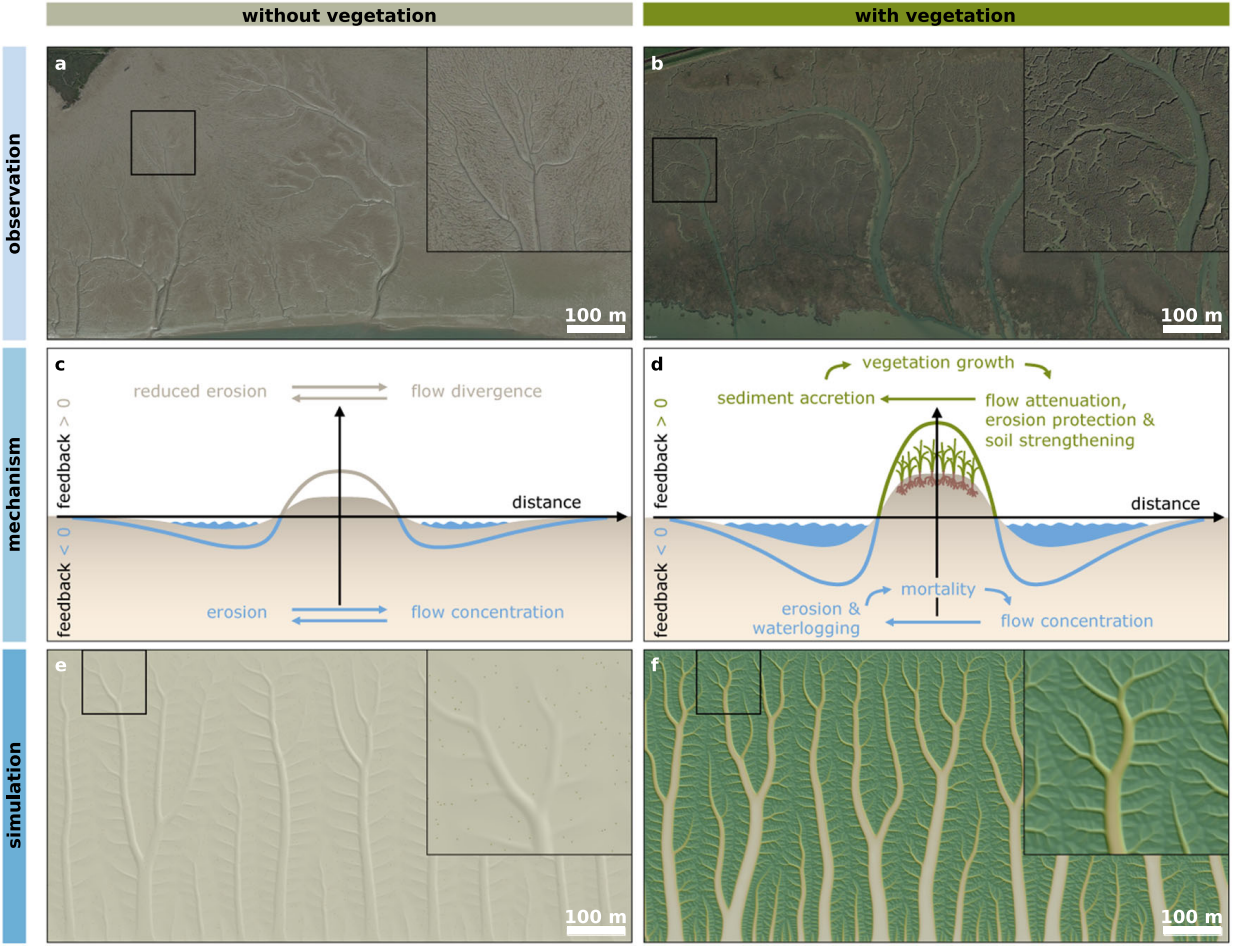
The influence of vegetation on tidal networks. **a**, **b** Branching tidal channel networks on an unvegetated tidal flat (51°22'3.71“N, 0°47'24.21“E) and a vegetated saltmarsh about 5.5 km seaward within the same estuary (51°21'47.96“N, 0°52'7.58“E); both aerial images dating from May 2018 and adapted from Google Earth Pro, © 2022 Google LLC. The larger square insets show a magnified view of the smaller squares, revealing the more complexly branching network in the vegetated compared to the unvegetated site. **c** Illustration of the abiotic channelization feedback, where water flow converges in local depressions, leading to further channel incision scouring and a reduction of erosion in between channels. This is a scale-dependent feedback, as the self-reinforcing feedback changes sign at distances further away from the center of flow divergence. **d** Vegetation amplifies this abiotic channelization feedback, leading to a biogeomorphic scale-dependent feedback. **e** In the absence of vegetation, the simulation model presented in this study generates branching networks, as illustrated in this shaded relief map (grey colors). **f** When vegetation amplifies the channelization feedbacks, a higher order of network branching occurs. In both e and f, the vegetation density map is shown in green colors; the shaded relief map is overlayed semi-transparently. See Supplementary Fig. 1 for the exact elevation and vegetation density maps. For both simulations, the bottom is an open outflow boundary; the other three boundaries are closed. Simulated time is 50 years. See also Table 3 for parameter settings. Source data are provided as a Source Data file.

Spatial patterning is intrinsic to natural systems, from individual organisms^1–3^ to entire landscapes^4–9^. Such spatial structuring can affect landscape functioning and increase ecosystem resilience to disturbances^9–16^. Tidal channel patterns in coastal wetlands are notable examples^4,12,17–20^, with geometries ranging from simple parallel ridges and runnels^12,19^ to complexly branching networks with multiple orders of channels (Fig. 1a, b). As tidal channels govern water fluxes^17^, their architecture ultimately determines the valuable ecosystem services that coastal wetlands provide for their highly populated hinterland^21,22^, such as nature-based mitigation of flood risks^23,24^. Furthermore, channel geometry strongly steers the resilience of wetlands to sea level rise^25–27^, as the supply and accretion of sediment is (apart from resuspension due to waves) largely conducted through channels^28,29^. It is therefore essential to understand the mechanisms underlying the morphological diversity, from simple to complex, of patterned ecosystems in general and of tidal networks in particular.

In the absence of vegetation, networks (particularly in terrestrial landscapes) branch due to a channelization feedback (Fig. 1c), where slight topographic depressions on gently sloping terrain attracts the water flow, thereby incising further and leading to flow divergence away from the areas in between channels^30^. Perpendicular to these primary channels, secondary topographic gradients emerge, which trigger secondary flows and lead to the formation of channel branches, a recursive process that can shape complexly branching networks. Similar feedbacks occur in tidal channel networks^20,31,32^, although modulated by the combination of ebb and flood flow directions^33^. The spacing between neighboring channels and the degree of channel branching are controlled by the rate at which channels are incised, relative to the rate at which topographic relief is smoothened out by topographic diffusion or soil creep^30,34,35^. With increasing ratio between incision and diffusion rates (known as the landscape Péclet number^34,35^), the spacing between parallel channels decreases and their banks steepen, leading to the incision of more channel branches^34,35^. In terrestrial settings, vegetation cover is typically considered to decrease runoff and increase soil diffusion through bioturbation, thereby reducing the Péclet number and thus network complexity^34,36–38^. A similar predictor for tidal networks, whose complexity is typically assumed to increase with vegetation presence^12,18,25^, as can also be observed in Fig. 1a, b, remains to be defined.

In coastal wetlands, sediment-stabilizing organisms (biofilms^12^, algal mats^19^ or plants^18,39^; from here on jointly referred to as vegetation) interact synergistically with the abiotic channelization feedback, thereby increasing network complexity (Fig. 1a, b), drainage efficiency and bank steepness^12,18,25,40,41^. Vegetation typically increases bed roughness^42–45^, erosion thresholds^12,46^ and sediment cohesion^40,41,46^, thereby locally decelerating water flow and promoting sediment accretion^47^. Water flow is deflected and accelerated around vegetated patches, causing sediment erosion and enhancing channel incision^12,18,39^ at some distance (Fig. 1d). On less eroded, elevated terrain, vegetation experiences better growth conditions^48,49^ and lower mortality from bed shear stress^50^ than in scour-induced depressions^51^. These biogeomorphic interactions trigger a scale-dependent feedback^12,18,19,39^, i.e., a self-reinforcing feedback between vegetation growth, flow attenuation and topography build-up that locally creates vegetated hummocks/ridges, but becomes self-inhibiting at some distance, creating unvegetated hollows/channels (Fig. 1d). Biological self-organization models based on such scale-dependent feedbacks generate single-scale, Turing-like patterns^3,6,11,12^, thus not explaining the multi-scale geometry of branching networks. More realistic biogeomorphic models that account for tidal dynamics and sediment transport are computationally costly, which makes it more difficult to capture many nested scales of channels. Overcoming these model-related challenges might advance our fundamental insight in the vegetation control on tidal channel network complexity.

Here we introduce a model for vegetation control on tidal drainage network formation. The model explicitly accounts for the biogeomorphic channelization feedback described above and illustrated in Fig. 1c, d, but has idealized sediment and tidal dynamics to increase our insight into how vegetation enhances channel formation at the many nested spatial scales of coastal wetlands. We first demonstrate how complexly branching tidal networks can develop in the absence of vegetation (Fig. 1e), but that vegetation boosts channel branching down to much finer spatial scales (Fig. 1f). We then explain the underlying mechanisms, linking the key vegetative factors to shifts in the balance between channel incision and topographic diffusion. Further, we use the model to demonstrate how growing relative vegetation importance leads to enhanced tidal network complexity, as well as improved ecosystem functioning and resilience. Finally, we support the qualitative simulation trends with network metrics from real-world wetlands.

## Results

### Model for network formation in vegetated coastal wetlands

To gain insight in the effect of vegetation on tidal network formation, we developed a model that explicitly accounts for the biogeomorphic feedback described above and schematized in Fig. 1d. Our model describes the coupled spatio-temporal dynamics of vegetation density, depth-averaged flow field and sediment bed elevation (“Methods”). The biogeomorphic feedback creates a scale-dependent erosion-sedimentation pattern, which amplifies any spatial differences in sediment bed elevation. This topography-building feedback is counteracted by topographic smoothing due to slope-driven sediment transport or soil creep^40^. Vegetation amplifies the abiotic channelization feedback (Fig. 1c) by attenuating and deflecting the flow with its above-ground biomass (e.g., algal filaments, plant stems) and by increasing the erosion threshold (e.g., EPS-secretion in biofilms, gluing together the sediment surface) with its below-ground biomass. These effects are included in our model through Manning’s roughness coefficient for vegetated beds (${n}_{{{{{{\rm{v}}}}}}}$) and the factor ${p}_{{{{{{\rm{E}}}}}}}$ by which vegetation reduces abiotic sediment erodibility. The degree of topographic smoothing is inversely related to soil cohesion, which is determined by abiotic factors (e.g., consolidation and grain size) as well as biotic factors (below-ground biomass, i.e., algal filaments or plant roots, creating a reinforcing network within the sediment, and EPS secretion). In the model, this effect is implemented through the factor ${p}_{{{{{{\rm{D}}}}}}}$ by which vegetation reduces abiotic soil diffusivity ${D}_{0}$. Earlier studies, focussing on erosive terrestrial landscapes, defined the landscape Péclet number, which is the ratio between channel incision and topographic diffusion rate^34,35^. In our model, the total incision rate is the product of abiotic incision processes (related to bed shear stress and abiotic sediment erodibility) and the biotic modulation of that (related to vegetation-induced roughness, ${n}_{{{{{{\rm{v}}}}}}}$, and erosion protection, ${p}_{{{{{{\rm{E}}}}}}}$). The total diffusion rate is the product of abiotic diffusion rate ${D}_{0}$ (related to abiotic sediment properties) and the biotic reduction of that (related to root-binding, ${p}_{{{{{{\rm{D}}}}}}}$). Our study aims at understanding how coastal wetland vegetation changes this incision-to-diffusion balance, and how this affects network complexity and functioning.

To allow for channel networks with an extensive range of spatial scales to be computed efficiently, some simplifying model assumptions are made. Although these assumptions make the model physically less realistic than existing models^26,31,39^, the associated reduction of model complexity allows to simulate and explain the qualitative effects of vegetation on channel network development over the wide range of spatial scales encountered in highly branching networks. First, assuming sheltered conditions, waves are neglected. Second, we model a steady continuous ebb discharge, assuming that the initial channel network formation is predominated by ebb flow drainage rather than by the flood flow, following earlier model studies^20,31,32^. This assumption avoids the computational demands of modelling full tidal water motion, which in turn allows rapid numerical solution of the mathematical equations for channel formation at many spatial scales over an extensive spatial domain. Third, we do not model advective sediment transport and instead assume that sediment supply is spatially homogeneous (in line with earlier studies^12,32^) but scaled with water depth. Although this simplification precludes the simulation of vegetation-induced sediment trapping, our study focusses on the vegetative controls on network development through flow deflection^18,43^, erosion protection^46^ and soil binding^40^, and we expect that the accretion pattern resulting from vegetation-induced erosion protection is qualitatively similar to that resulting from vegetation-induced sediment trapping. Constant drainage flow and fixed bed elevation at the outflow boundary ensure that channels can keep being incised and prevents infilling. Bed elevation is computed from a simplified balance between local sedimentation, erosion and slope-driven soil creep^40^. The latter is modelled as a diffusive process, in line with earlier model studies^32,40^. Finally, it is assumed that vegetation, by increasing sediment cohesion with its below-ground biomass and/or EPS, locally reduces soil creep^32^. The model is fully explained in the “Methods” section.

Despite our model simplifications, the simulated emergence of the complex, multi-scale patterns and the effect of vegetation therein (Fig. 1e, f, Supplementary Fig. 1, Supplementary Fig. 2) show strong qualitative resemblance with many characteristics of real-world channel network development. Starting from a flat, horizontal bed with sparse and randomly dispersed vegetation tussocks (one grid cell per tussock), a sedimentary bed slope develops as a result of continuous sedimentation and an erosional gradient induced by the ebb flow. As water flow is slowed down by vegetation, flow concentrates in the open areas in between vegetation patches. This leads to sedimentary hummock formation in vegetated patches and increased erosion in between vegetated patches, resulting in the development of flow channels (Fig. 1d). A network of shallow, braiding channels emerges (Supplementary Fig. 2). As time progresses, these parallel streams merge into fewer, deeper channels, in line with earlier studies^52^. The network becomes increasingly complex over time, i.e., the development of main (first-order, following Hack’s stream order) channels is followed by the emergence of increasingly fine-scaled (second-, third-order etc.) side-branches, resulting in a multi-scale channel network. Typical aspects of the simulated development agree qualitatively with observations, e.g., channel incision in between expanding vegetation^18,39^, increased channel bank steepness in vegetated compared to unvegetated networks^40^, and the development of increasingly high-order side branches^17,31^. Similar developmental stages are simulated in the abiotic case (Supplementary Fig. 2), but vegetation leads to a significantly more branched creek network (Fig. 1e, f).

### Vegetation enables channel branching down to finer scales

**Fig. 2:**
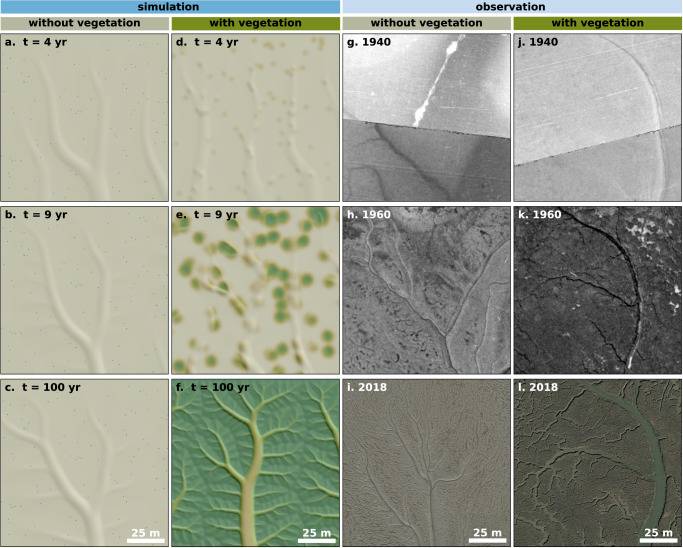
Vegetation enables recursion of the channelization feedback down to finer spatial scales. **a**–**c** Simulated development of a multi-scale regular channel network in the absence of vegetation, due to recursion of the abiotic scale-dependent feedback in Fig. 1c. The same model setup is used as in Fig. 1e and the same zoomed-in region is shown here. **d**–**f** With vegetation, the model predicts finer-scale channel braches, as vegetation amplifies the scale-dependent feedback, see Fig. 1d. As a result, the nesting of regularly-spaced side-channels continues down to finer scales (higher Hack stream order). The same model setup as in Fig. 1f is used and the same zoomed-in region is shown. For all panels a-f, vegetation density is shown in green colors and topography (shaded relief map, in grey colors) is overlayed semi-transparently; see Supplementary Fig. 1 for the topography and vegetation density maps, and Table 3 for parameter settings. Channel network development observed in aerial images of (**g**–**i**) an unvegetated tidal flat (51°22'2.76“N, 0°47'5.91“E) and (**j**–**l**) a vegetated tidal marsh (51°21'52.54“N, 0°51'38.55“E), both near Leysdown-on-Sea, UK; these areas are identical to the zoomed-in regions in Fig. 1e, f. In the unvegetated site, no significant or persistant vegetation cover was observed in aerial images throughout the period 1940–2018; in the vegetated site, clear and persistant vegetation cover was observed in aerial images from 1960 onwards, as indicated in the darker color of panel k. Although fine-scale and shallow ridge-runnel-like patterns are observed in the unvegetated site, the actual channel network is more pronounced and complexly branching in the vegetated site. Note that panels g, h, j, k (copyright © 2023 Kent County Council) are black-and-white photos, and that the diagonal lines in panels g,j are the borders between adjacent photos. The white elongated mark in panel g seems an artefact in the aerial photo. All aerial images adapted from Google Earth Pro, © 2022 Google LLC, and dating from December 1940, December 1960 and May 2018, respectively. Source data are provided as a Source Data file.

To understand how vegetation creates more complex channel networks, we zoom in on the recursive behaviour of the channelization feedback (Fig. 2). The model shows that, at first, the development of the main topographic gradient (from top to bottom in Fig. 2a, d) induces water drainage flow and hence triggers the channelization feedback (Fig. 1c), which creates a single-scale pattern of regularly spaced channels (1st-order, according to Hack’s stream ordering system; Fig. 2a, d). Since vegetation has not yet colonized the tidal flat significantly at this stage, the channelization feedback is mainly abiotic. Perpendicular to these channels, a secondary elevational gradient develops (left-right in Fig. 2a, d, i.e., following the bank slopes of 1^st^-order channels), which again invokes a scale-dependent feedback that leads to the incision of second-order branches (2nd-order, Fig. 2b, e). Vegetation colonization starts to have a significant effect at this stage, leading to channel incision between vegetation tussocks, steeper channel banks and more pronounced 2^nd^-order branches (Fig. 2e) compared to the unvegetated case (Fig. 2b).

With ongoing vegetation development, the biotic effect on channel bank steepness and network complexity becomes increasingly apparent (Fig. 2c, f). In the absence of vegetation, channel banks are not steep enough and the abiotic channelization feedback (Fig. 1c) is not strong enough to incise many finer-scale channels. Vegetation, however, amplifies the channelization feedback (Fig. 1d), leading to steeper slopes and allowing the recursive feedback to form even finer-scale (3rd-order, and so on) channels, giving rise to an increasingly complex pattern that consists of single-scale regular patterns, nested within one another to form a multi-scale regular pattern. The recursive channelization feedback known from terrestrial geomorphology^34,35^ can thus be applied to coastal landscapes as well, but the effect of vegetation in coastal wetlands is markedly different from erosive terrestrial landscapes, where vegetation is typically associated to reduce runoff and increase soil diffusion due to bioturbation^34,36–38^. Our detailed model analyses (Supplementary Fig. 3) show that in coastal wetlands, vegetation increases scour around vegetated patches and into channels (as shown by increasing vegetative roughness, ${n}_{{{{{{\rm{v}}}}}}}$), amplifies the elevation difference between vegetated platform and bare channels (as shown by increasing erosion protection, ${p}_{{{{{{\rm{E}}}}}}}$) and increases bank slopes (as shown by increasing the vegetative potential to reduce topographic diffusion, ${p}_{{{{{{\rm{D}}}}}}}$). While these individual processes are known from previous studies^12,18,40,43,45,46^, a coherent framework to explain how vegetation creates more complex networks^12,18,25^ was lacking. We show how all the key vegetation effects (modelled through ${n}_{{{{{{\rm{v}}}}}}}$, ${p}_{{{{{{\rm{E}}}}}}}$ or ${p}_{{{{{{\rm{D}}}}}}}$) increase the ratio between channel incision and topographic diffusion rate (the landscape Péclet number^34,35^) or retain this ratio high until finer scales of channel nesting.

We observe similar sequences of channel nesting, and an increase in channel branching in real-world saltmarshes (Fig. 2j–l) compared to nearby unvegetated sites (Fig. 2g–i). More real-world examples are shown in Supplementary Fig. 9, which includes observations from a tidal flat (“Methods”), where the scale-dependent feedback is induced by filamentous algal mats. All examples shown how the unvegetated channel geometry develops quickly, but the incision of finer scale side-branches occurs together with the colonization by vegetation. Although some higher-order channels may already arise in parts of the wetland even in the absence of vegetation while lower-order channels are still developing in other parts, network refining (i.e., the nesting of increasingly smaller channels, with higher Hack stream orders) in synchrony with vegetation establishment is the dominant tendency.

Further model analysis reveals that, once the vegetation-induced scale-dependent feedback loses its strength, the sequence of continuous network refining stops (Supplementary Fig. 4). The fact that vegetation in coastal wetlands preferentially grows on elevated terrain and not in channels, is key to the scale-dependent biogeomorphic feedback (Fig. 1d). We find that, in the finest channels, water depth and flow speeds become small enough for vegetation to colonize these channels, such that the biogeomorphic scale-dependent feedback is steeply reduced in strength (Supplementary Fig. 4d). Consequently, the degree of channelization of a given drainage area decreases, and the power-law relation^53^ between channel length and drainage area breaks down (Supplementary Fig. 4a–c). This is a likely explanation for the similar scale break in the scaling relations observed in real-world wetlands^20^. In other words, vegetation can keep amplifying the abiotic scale-dependent feedback (Fig. 1c, d), thereby triggering a next level of network refinement far beyond the smallest scale where the abiotic feedbacks cease, until finally the differences in topography and flow speed become so subtle that vegetation does not experience differences in growth conditions anymore and thus cannot amplify the abiotic channelization feedback any further.

### Enhanced network functioning and ecosystem resilience

**Fig. 3:**
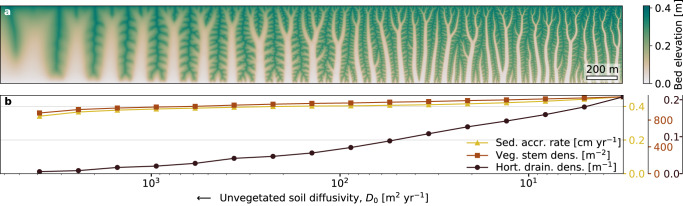
Effect of relative biogeomorphic feedback strength on network complexity and coastal wetland functioning. **a** Simulated sediment bed level along a spatial gradient in soil cohesion (cohesion increases towards the right). Soil cohesion is varied independently from vegetation presence, i.e., it is the abiotic cohesion, inversely related to model parameter ${D}_{0}$ (unvegetated soil diffusivity, or the magnitude of soil creep). Lower diffusion translates to increased relative biogeomorphic feedback strength. Shown networks after 50 years of development. See Table 3 for parameter settings. **b** Associated changes in coastal wetland functioning, i.e., sediment accretion rate (abbreviated as sed. accr. rate, spatially averaged and averaged over the 50 years of development), channel network efficiency (Hortonian drainage density, abbreviated as Hort. drain. dens., in the state after 50 years) and marsh productivity (spatially averaged vegetation density, abbreviated as veg. stem dens., in the shown state after 50 years). The calculations in Fig. 3b are done using individual model simulations with a fixed ${D}_{0}$-value, as indicated by the 16 horizontal positions of the data points along the ${D}_{0}$-gradient. Going from right to left (from low to high ${D}_{0}$), these 16 simulations are numbered SIM_2 to SIM_17 (see Supplementary Fig. 6). The simulation with the maximal ${D}_{0}$-value along the ${D}_{0}$-gradient is numbered SIM_18, but the network in this simulation is so wide and smooth that a channel network could not be extracted. Source data are provided as a Source Data file.

Further model analysis demonstrates how biogeomorphic feedback strength directly controls the spatial complexity of channel networks (Fig. 3a) and, with that, coastal wetland functioning (Fig. 3b). We chose to modify the relative strength of the biogeomorphic feedback by adjusting abiotic soil cohesion (inversely related to ${D}_{0}$), which reflects physical parameters such as grain size and consolidation. A lower abiotic soil diffusion rate (lower ${D}_{0}$) implies a reduction in topographic smoothing, such that the vegetative factors (modelled through parameters ${n}_{{{{{{\rm{v}}}}}}},{p}_{{{{{{\rm{E}}}}}}}$ and ${p}_{{{{{{\rm{D}}}}}}}$, see Supplementary Fig. 3) have a relatively stronger effect on the incision-vs-diffusion balance. Therefore, a decrease in topographic smoothing (${D}_{0}$) effectively leads to an increase in the relative strength of the biogeomorphic feedback. Indeed, a decrease in ${D}_{0}$ leads to a more strongly branching network, just as an increase in the combined vegetative effects (${n}_{{{{{{\rm{v}}}}}}},{p}_{{{{{{\rm{E}}}}}}}$ and ${p}_{{{{{{\rm{D}}}}}}}$) does (Supplementary Fig. 3). Although decreasing ${D}_{0}$ also decreases the overall channel spacing (a trend not clearly observed when increasing the three vegetation parameters), the single control parameter ${D}_{0}$ does qualitatively synthesize the combined effect of vegetation (${n}_{{{{{{\rm{v}}}}}}},$ ${p}_{{{{{{\rm{E}}}}}}}$, ${p}_{{{{{{\rm{D}}}}}}}$) on channel complexity, which is the focus of our study. A relatively weak biogeomorphic feedback (i.e., at low soil cohesion) results in a low-complexity network consisting of low-order channels with gentle bank slopes, whereas a relatively strong biogeomorphic feedback (i.e., at high soil cohesion) yields a high-complexity network with more frequently branching channels and steeper bank slopes (Fig. 3a), in line with observations of earlier studies^18,25,40^.

Higher relative biogeomorphic feedback strength due to increased abiotic soil cohesion (decreased ${D}_{0}$) in the model also results in a more efficient drainage network, higher mean vegetation densities and faster mean sediment accretion (Fig. 3b). The expansion of the drainage network is also observed when jointly increasing the biotic parameters (${n}_{{{{{{\rm{v}}}}}}},{p}_{{{{{{\rm{E}}}}}}}$, ${p}_{{{{{{\rm{D}}}}}}}$; see Supplementary Fig. 3). Because of this expansion of channelized area, the domain-averaged vegetation density and sediment accretion rate do not show the same clear increase as we found in Fig. 3b. However, vegetated areas become more densely vegetated and gain elevation, while the (already low) vegetation density in channels becomes even lower and the channels become deeper, when the biotic parameters are increased. As a result, the bed elevation and vegetation pattern become more strongly correlated (Supplementary Fig. 3), which has previously been shown to indicate a stronger vegetative control on wetland morphology^19^. Our simulations imply that, with boosted biogeomorphic feedback strength, marsh platforms will accrete more rapidly while the channel network deepens and becomes more extensive. We expect that this improves wetland resilience to sea level rise, which strongly depends on proper drainage^48,49,54^ and sediment accretion^28,29^. Moreover, the simulated increase in vegetation cover and elevation of the marsh platform translates to increased bed roughness over the marsh, which is expected to enhance storm surge attenuation by the wetland^21,22,55^. Finally, networks with a higher number of channel branches are expected to be more resilient to perturbations^27^. Our model suggests a pivotal role for vegetation-induced self-organization in shaping wetlands with improved ecosystem functioning and resilience, hence providing a clear hypothesis for future field studies.

### Qualitative comparison of modelled and real-world networks

**Fig. 4:**
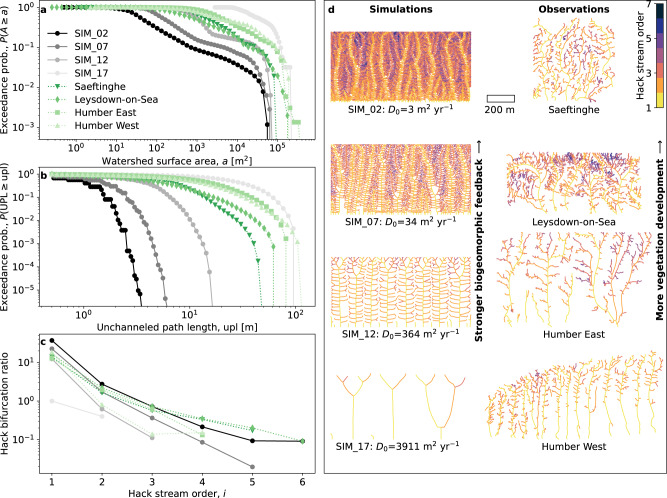
Comparison between the geometric characteristics of simulated and real-world tidal channel networks, shown in order of decreasing relative biogeomorphic feedback strength (simulations) and decreasing degree of vegetation establishment (real-world). **a** Probability P (abbreviated as Exceedance prob.) that watershed surface area A exceeds a, for a selection of four simulated and four real-world tidal channel networks. The simulation numbering and unvegetated soil diffusivity (${D}_{0}$) correspond with the simulations shown in Fig. 3b, i.e. higher simulation number corresponds to lower soil strength and thus lower relative biogeomorphic feedback strength. See Table 3 for parameter settings of the model simulations. Watershed surface areas were computed at equidistant (0.5 m) points along the entire stream skeleton. The degree of vegetated marsh development ranges from well-developed (Saeftinghe and Leysdown-on-Sea) to newly developed (Humber East) to poorly developed (Humber West); see “Methods” for details. **b** Probability P that unchanneled path length UPL exceeds upl, for the same simulated and real-world channel networks. Unchanneled path lengths were computed at each unchanneled grid cell. **c** Bifurcation ratios of these four simulated and four real-world networks. Hack’s stream ordering is used, and bifurcation ratios are computed as ${r}_{i}={n}_{i+1}/{n}_{i}$, with bifurcation ratio ${r}_{i}$ at Hack stream order i, and ${n}_{i}$ the number of streams (or individual subbasins) of this order. **d** Stream skeletons of the simulated and real-world networks of Fig. 4a–c. Colors denote Hack stream order. The scale bar applies to all eight networks. The digital terrain models used to extract these eight networks as well as the corresponding maps of unchanneled path length are shown in Supplementary Fig. 5 (real-world networks) and Supplementary Fig. 6 (simulated networks). Source data are provided as a Source Data file.

Despite the simplified nature of our model, several fundamental geometric properties of the simulated networks are qualitatively comparable to the real-world networks (Fig. 4). We considered four different field sites: a tidal marsh in the Drowned Land of Saeftinghe (the Netherlands), the vegetated tidal marsh near Leysdown-on-Sea (United Kingdom) and two sections (in the east and in the west) of a tidal marsh in the Humber estuary near Hull (United Kingdom). The tidal channel networks of these field sites (Fig. 4d) were extracted from digital terrain models (Supplementary Fig. 5), and several geometric properties were calculated (see the “Methods” section for details). The same approach was applied to four simulated channel networks, which were chosen at four regular intervals along the entire ${D}_{0}$-gradient in Fig. 3. We find that the exceedance probabilities of watershed surface areas (Fig. 4a) and unchanneled path lengths (Fig. 4b) follow a non-scale-free relationship, both for real-world and simulated networks. This pointed absence of power laws is considered one of the hallmarks of tidal channel networks^31,56,57^. Furthermore, channel bifurcation ratios decrease consistently with increasing Hack stream order, both for real-world and simulated networks (Fig. 4c). The bifurcation ratio is here calculated using Hack stream orders, and hence computed as the number of streams of one order higher (i.e., more fine-scaled) compared to the current order. This monotonous decrease of bifurcation ratio with increasingly fine spatial scales is in line with our hypothesis of scale-dependent feedback recursion, since the scale-dependent feedback strength (e.g., erosive power) is expected to decrease with refining spatial scales (see also Supplementary Fig. 4).

Although the absolute numbers differ, simulated and real-world networks show qualitatively similar trends with increasing biogeomorphic feedback strength. The four field sites included here have different degrees of marsh development (see “Methods”): Saeftinghe and Leydown-on-Sea developed into vegetated marshes long ago, whereas the two sites in the Humber estuary have developed from a tidal flat into a vegetated marsh more recently. Furthermore, in the Humber East site more vegetation development took place compared to the Humber West site. This is reflected in the channel network statistics: the older marshes (Saeftinghe, Leysdown-on-Sea) have smaller average drainage areas, shorter average unchanneled path lengths and finer levels of branching (Fig. 4) and higher Hortonian drainage densities (Supplementary Fig. 7) than the younger marshes (Humber East and West). This trend agrees qualitatively with the simulated networks, moving from high to low ${D}_{0}$, i.e., from low to high relative biogeomorphic feedback strength. Another way of comparing simulated and real-world networks is to compare the similarity between two probability distributions using the two-sample Kolmogorov-Smirnov test for goodness of fit (Supplementary Fig. 7). Indeed, when comparing the probability distributions of drainage area and unchanneled path length, the more developed real-world marshes show their highest goodness of fit when compared with lower-${D}_{0}$ simulated networks, whereas the younger marshes agree better with higher-${D}_{0}$ networks. Although simulation parameters such as ${D}_{0}$ need to be calibrated further in future studies to find better quantitative agreement between model and field, the simulated trends (i.e., the finer scale of channel nesting in the presence of vegetation) agree qualitatively with observations.

## Discussion

In this study we have demonstrated how vegetation allows channel branching to continue down to finer spatial scales, by locally reducing topographic diffusion and enhancing channel incision. By linking these vegetative effects to the ratio between incison and diffusion rates, a ratio known as the landscape Péclet number and used to predict the degree of terrestrial valley branching^34,35^, we provide a mechanistic framework to interpret the many previous studies that identified a strong effect of vegetation on the development of channelized coastal wetlands^18,25,39^. Interestingly, our study highlights that coastal wetland vegetation typically increases the incision-to-diffusion ratio and thus promotes channel branching and network complexity, which is opposite to the vegetation effects described for terrestrial channel networks^36–38^. The model framework presented here may also be insightful for the interpretation of the synchronous complexification of sedimentary landscapes and biostabilizing organisms through geological time^58^, as the evolution of biostabilizers with increasingly pronounced roots may have decreased topographic diffusion and hence strengthened biogeomorphic feedbacks. Finally, although the formation of multi-scale regular patterns (i.e., branching channel patterns) has been long recognized in terrestrial geomorphology^30,34,35^, scale-dependent feedbacks in the field of biological self-organization are typically linked to single-scale regular patterns (e.g., Turing patterns)^3,6,11,12^ whereas multi-scale ecosystem patterns are typically explained by a combination of multiple feedback mechanisms^13,59^. Our study emphasizes that, although abiotic feedbacks (Fig. 1c) may often initiate landscape patterning, biota often further amplify this abiotic feedback^19,41^ (Fig. 1d), leading to recursive dynamics and thus allowing complex multi-scale patterns to arise from a single-scale feedback process.

In an era in which coastal lowlands are getting increasingly populated and at the same time become progressively exposed to the risk of flooding due to sea level rise and amplified storm intensity, it is of paramount importance to explore the nature-based flood-mitigating effects of coastal wetlands such as tidal flats, marshes and mangrove forests^23,24,55^. In this study, we have shown through modelling that vegetation boosts the abiotic feedback process that generates branching channel networks, leading to enhanced accretion and vegetation growth on the marsh platform, as well as more extensive drainage channel networks. These findings imply that biogeomorphic self-organization could increase the potential of coastal wetlands to keep up with sea level rise^19,28,48,54^ (i.e., due to improved drainage, sediment supply through the channel network, and vertical marsh accretion) and enhanced storm surge attenuation capacity^21,22,55^ (i.e., due to higher marsh platforms with increased vegetative roughness). Future field studies are needed to support these findings. Our work suggests that preserving natural landscape complexity in existing coastal wetlands and facilitating biogeomorphic feedbacks in wetland restoration projects is vital to ensure resilient coastal wetland ecosystems with all the ecosystem services they can provide to coastal communities worldwide.

## Methods

### Model of vegetation control on wetland channel networks

A model is developed to study how tidal channel networks emerge from the scale-dependent feedbacks that result from interactions between water flow, sedimentary processes and vegetation, and particularly how vegetation affects these feedbacks. This model is referred to as SFERE (Scale-dependent Feedback Recursion) and is publicly available online^60,61^. The focus area, spanning in cross-shore (*x*-coordinate) and along-shore (*y*-coordinate) directions, initially is topographically flat and with sparse, randomly dispersed vegetation tussocks (each tussock spans one grid cell; vegetation establishment only occurs at the start of the simulation, in roughly ${p}_{{{{{{\rm{est}}}}}}}=0.2\%$ of all grid cells). The area is bordered by topographic upland or dikes on three of its lateral boundaries; the fourth boundary represents the area’s connection to the main tidal channel (Supplementary Fig. 8). The model builds on an earlier, strongly idealized, one-dimensional (*y*-direction only) model for the self-organization of diatom patterns on tidal flats^12^. This previous model is here extended into two dimensions and equipped with more realistic equations for hydrodynamic, sedimentary and biotic processes. The values of all parameters (as discussed hereafter), as well as specification of settings per Figure shown, is given in Tables 1–3.

**Table 1:** Overview of dependent and independent variables in the model

Variable	Unit	Interpretation
*x*	m	Horizontal coordinate (shoreward, i.e. down-slope)
*y*	m	Horizontal coordinate (alongshore, i.e. cross-slope)
*t*	s	Time
*B*	m^−2^	Vegetation stem density
*h*	m	Water layer thickness
*S*	m	Sediment layer thickness
*u*	m s^−1^	Depth-averaged flow component in *x*-direction
*v*	m s^−1^	Depth-averaged flow component in *y*-direction
*η*	m	Water surface elevation
${C}_{{{{{{\rm{z}}}}}}}$	m^1/2^ s^−1^	Chézy coefficient
${D}_{S}$	m^2^ s^−1^	Sediment diffusivity (topographic diffusivity)
${\tau }_{{{{{{\rm{b}}}}}}x},{\tau }_{{{{{{\rm{b}}}}}}y},{\tau }_{{{{{{\rm{b}}}}}}}$	N m^−2^	Bed shear stress in *x*- and *y*-directions and absolute value

**Table 2:** Overview of the parameters used in the default model simulation, i.e. Fig. 1f

Parameter	Value/unit	Interpretation	Ref.
${l}_{x}$	512 m	Domain length in *x*-direction	-
${l}_{y}$	1024 m	Domain width in *y*-direction	-
${n}_{x}$	1024 [-]	Number of grid cells in *x*-direction	-
${n}_{y}$	2048 [-]	Number of grid cells in *y*-direction	-
$\Delta {{{{{\rm{x}}}}}}$	${l}_{x}/{n}_{x}=0.5{{{{{\rm{m}}}}}}$	Grid resolution in *x*-direction	-
$\Delta {{{{{\rm{y}}}}}}$	${l}_{y}/{n}_{y}=0.5{{{{{\rm{m}}}}}}$	Grid resolution in *y*-direction	-
$\Delta {{{{{\rm{t}}}}}}$	0.0125 s	Time step size	-
Φ	44712 s [T_M2_]^−1^	Morphological acceleration factor. Calculated as the number of seconds in one M2-tidal period T_M2_.	^12^
${t}_{{{{{{\rm{E}}}}}},{{{{{\rm{M}}}}}}2}$	35290 [T_M2_]	End time (i.e., simulated time), in M2-tidal periods	-
${t}_{{{{{{\rm{E}}}}}},{{{{{\rm{yr}}}}}}}$	50 yr	End time (i.e., simulated time), in years	-
${D}_{0}$	10^−7^ m^2^ s^−1^	Sediment diffusivity in the absence of vegetation	^32^
${D}_{B}$	6.0*10^−9^ m^2^ s^−1^	Vegetation diffusivity (~clonal expansion rate)	^18^
${D}_{{{{{{\rm{U}}}}}}}$	0.5 m^2^ s^−1^	Turbulent eddy viscosity	e
${E}_{S}$	2.5*10^−4 ^s m^−2^	Sediment erosion rate (in absence of vegetation and at unit bed shear stress)	d
${E}_{B}$	1.0*10^−5 ^s m^−2^	Vegetation erosion rate (at unit bed shear stress)	e
*g*	9.81 m s^−2^	Gravitational acceleration constant	-
${H}_{0}$	0.02 m	Initial water layer thickness	^12^
${H}_{{{{{{\rm{c}}}}}}}$	10^−3 ^m	Critical water layer thickness that always remains	e
${H}_{{{{{{\rm{in}}}}}}}$	1.0*10^−5 ^m s^−1^	Water input (tidally averaged discharge velocity)	d
*k*	1500 m^−2^	Vegetation carrying capacity (max. stem density)	^29^
${n}_{{{{{{\rm{b}}}}}}}$	0.016 s m^−1/3^	Manning’s roughness coeff. for bare sediment	^66^
${n}_{{{{{{\rm{v}}}}}}}$	0.2 s m^−1/3^	Manning’s roughness coeff. for vegetated sediment	^66^
${p}_{{{{{{\rm{D}}}}}}}$	0.99 [-]	Fraction by which sediment diffusivity is reduced when vegetation is at carrying capacity	^32^
${p}_{{{{{{\rm{E}}}}}}}$	0.9 [-]	Fraction by which sediment erosion is reduced when vegetation is at carrying capacity	^12^
${p}_{{{{{{\rm{est}}}}}}}$	0.002 [-]	Probability of vegetation seedling establishment	e
${Q}_{{{{{{\rm{q}}}}}}}$	0.02 m	Water layer thickness at which veg. growth is halved	e
${Q}_{{{{{{\rm{s}}}}}}}$	6.0*10^−4 ^m	Water layer thickness at which sediment input is halved	e
*ρ*	1000 kg m^−3^	Reference water density	-
*r*	3.2*10^−8 ^s^−1^	Intrinsic plant growth rate (=1 year^−1^)	^18^
${S}_{{{{{{\rm{in}}}}}}}$	5.0*10^−9 ^m s^−1^	Maximal sediment input rate	d

For dimensionless parameters, the (absence of) units is denoted as [-]. Column Ref. refers to the references (numbered according to the reference lists) from which parameter values were deduced. Empirically tuned parameters are denoted as e. Derived parameters are denoted as d; derivations of these parameter values are explained in the “Methods” section.

**Table 3:** Overview of parameters used for each shown simulation result

Figure	Changes compared to the default model
Fig. 1f, Supplementary Fig. 1b, d, f, h	Default model run; see Tables 1 and 2 for parameter settings.
Fig. 2d–f, Supplementary Fig. 2e–h	As the default model, but shown at different values of ${t}_{{{{{{\rm{E}}}}}},{{{{{\rm{yr}}}}}}}$, as indicated in the figures themselves.
Fig. 3a	As the default model, but with a spatial gradient in ${D}_{0}$, ranging from ${D}_{0,\min }=1.0*{10}^{-7}{{{{{{\rm{m}}}}}}}^{2}{{{{{{\rm{s}}}}}}}^{-1}$ to ${D}_{0,\max }=2.0*{10}^{-4}{{{{{{\rm{m}}}}}}}^{2}{{{{{{\rm{s}}}}}}}^{-1}$, a 4x wider domain (*l*_*y*_ = 4096 m; *n*_*y*_ = 8192) and a smaller time step (Δt = 0.005 s).
Fig. 3b, Supplementary Fig. 6, Supplementary Fig. 7	As the default model, but 17 different simulations, each with a different value of ${D}_{0}$. These ${D}_{0}$-values are chosen equidistantly along the gradient in Fig. 3a, according to Eq. (22) in the “Methods” section. Here, also a smaller time step (Δt = 0.005 s) is used.
Fig. 4	Four simulations selected from the 17 simulations in Fig. 3b; see the figure captions for specification.
Fig. 1e, Supplementary Fig. 1a, c, e, g	As the default model, but vegetation dynamics deactivated $\left(\frac{\partial B}{\partial t}=0\right)$.
Fig. 2a–c, Supplementary Fig. 2a–d	As Fig. 1e, but shown at different values of ${t}_{{{{{{\rm{E}}}}}},{{{{{\rm{yr}}}}}}}$, as indicated in the figures themselves.
Supplementary Fig. 3	As Fig. 3b, but with different (combinations of) values for ${D}_{0}$, ${p}_{{{{{{\rm{D}}}}}}}$, ${p}_{{{{{{\rm{E}}}}}}}$ and ${n}_{{{{{{\rm{v}}}}}}}$. See the figure itself for these parameter values.
Supplementary Fig. 4	As the default run, but with doubled time step size, i.e. Δt = 0.025 s.

In this study it is assumed that ebb flow is the dominant hydrodynamic component, in line with earlier studies^20,31,32^. Waves are neglected, assuming a sheltered tidal marsh. Water flow is prescribed by the depth-averaged shallow water equations^62^. The continuity equation, i.e.

1 $$\frac{\partial \eta }{\partial t}=-\frac{\partial \left({uh}\right)}{\partial x}-\frac{\partial \left({vh}\right)}{\partial y}$$

prescribes how the water surface elevation, *η*, changes in time, *t*, due to convergence or divergence of water flow. The water layer has local thickness *h* and its depth-averaged flow velocities are expressed in a shoreward (down-slope) component *u* and an alongshore (cross-slope) component *v* (Supplementary Fig. 8). In order to simplify the model as much as possible, which allows faster computations and hence higher spatial resolution, it is assumed that ebb discharge is uniform in space and time. Therefore, it is assumed that the entire tidal prism drains away steadily over the course of one tidal cycle. This yields a spatially and temporally constant water source term, ${H}_{{{{{{\rm{in}}}}}}}$, that is added to the continuity equation (following earlier studies^12^). ${H}_{{{{{{\rm{in}}}}}}}$ is estimated as the mean rate at which the tidal prism drains away over a tidal period, and is hence related to the tidal range (see further on in the “Methods” section). Moreover, it is assumed that the temporal development of bottom topography *S* is relatively slow compared to variations in water layer thickness, i.e.

2 $$\frac{\partial \eta }{\partial t}=\frac{\partial \left(h+S\right)}{\partial t}\, \approx \, \frac{\partial h}{\partial t}$$

such that the continuity equation is written as:

3 $$\frac{\partial h}{\partial t}=-\frac{\partial \left({uh}\right)}{\partial x}-\frac{\partial \left({vh}\right)}{\partial y}+{H}_{{{{{{\rm{in}}}}}}}$$

A simple wetting-drying algorithm is employed, to ensure that the shallow water equations can be prescribed, even in regions of the intertidal area that accrete so much sediment that they become non-submerged. This is done by employing a thin film algorithm^63^, which imposes that water layers never become thinner than a prescribed critical layer thickness ${H}_{{{{{{\rm{c}}}}}}}$:

4 $$h=\max \left(h,\, {H}_{{{{{{\rm{c}}}}}}}\right)$$

The shallow water flow field is calculated from the momentum equations^62,64^:

5 $$\frac{\partial u}{\partial t}=-g\frac{\partial \left(h+S\right)}{\partial x}-u\frac{\partial u}{\partial x}-v\frac{\partial u}{\partial y}-\frac{{\tau }_{{{{{{\rm{b}}}}}}x}}{\rho h}+\nabla \left({D}_{{{{{{\rm{U}}}}}}}\nabla u\right)$$

6 $$\frac{\partial v}{\partial t}=-g\frac{\partial \left(h+S\right)}{\partial y}-u\frac{\partial v}{\partial x}-v\frac{\partial v}{\partial y}-\frac{{\tau }_{{{{{{\rm{b}}}}}}y}}{\rho h}+\nabla \left({D}_{{{{{{\rm{U}}}}}}}\nabla v\right)$$

Equations (5) and (6), respectively, prescribe how depth-averaged cross-shore and along-shore flow velocities change in time due to pressure gradients (with gravitational acceleration constant *g* and sedimentary bed elevation *S*), advection of momentum, bed friction (with ${\tau }_{{{{{{\rm{b}}}}}}x}$ and ${\tau }_{{{{{{\rm{b}}}}}}y}$ the bed shear stress components in *x*- and *y*-direction, respectively, and water density *ρ*) and turbulent mixing (with $\nabla=\left(\frac{\partial }{\partial x},\frac{\partial }{\partial y}\right)$ and horizontal eddy viscosity ${D}_{{{{{{\rm{U}}}}}}}$). The bed shear stress components and their absolute value (${\tau }_{{{{{{\rm{b}}}}}}}$) are given by:

7 $$\frac{({\tau }_{{{{{{\rm{b}}}}}}x},{\tau }_{{{{{{\rm{b}}}}}}y},{\tau }_{{{{{{\rm{b}}}}}}})}{\rho }=\frac{g}{{{C}_{{{{{{\rm{z}}}}}}}}^{2}}\sqrt{{u}^{2}+{v}^{2}}\left(u,v,\sqrt{{u}^{2}+{v}^{2}}\right)$$

where the Chézy coefficient of the bed, ${C}_{{{{{{\rm{z}}}}}}}$, is given by Manning’s formulation^65^:

8 $${C}_{{{{{{\rm{z}}}}}}}=\frac{1}{n}{h}^{\frac{1}{6}}$$

Manning’s *n*, the bed roughness coefficient, is higher for vegetated bed than for bare sediment^66,67^ and is here assumed to increase linearly with vegetation stem density *B*, i.e.

9 $$n={n}_{{{{{{\rm{b}}}}}}}+\left({n}_{{{{{{\rm{v}}}}}}}-{n}_{{{{{{\rm{b}}}}}}}\right)\frac{B}{k}$$

where ${n}_{{{{{{\rm{b}}}}}}}$ and ${n}_{{{{{{\rm{v}}}}}}}$ are roughness coefficients for bare and vegetated beds, respectively, and *k* is the vegetation carrying capacity. Suspended sediment concentrations are assumed to be high enough, such that sediment availability is not limited by the lateral distance to channels. Moreover, the model focusses on vegetative effects on tidal network development, where trapping of suspended sediment by vegetation is expected to result in deposition patterns that are qualitatively similar to those arising from vegetation-induced bed roughness and erosion protection. Hence, to further speed up the calculations, lateral advective sediment transport is neglected. Therefore, elaborating on earlier model studies^12^, the sedimentary bed elevation *S* is prescribed by a simplified balance between sedimentation, erosion and slope-driven sediment transport:

10 $$\frac{\partial S}{\partial t}={S}_{{{{{{\rm{in}}}}}}}\frac{{h}_{{{{{{\rm{e}}}}}}}}{{Q}_{{{{{{\rm{s}}}}}}}+{h}_{{{{{{\rm{e}}}}}}}}-{E}_{S}\left(1-{p}_{{{{{{\rm{E}}}}}}}\frac{B}{k}\right)S\frac{{\tau }_{{{{{{\rm{b}}}}}}}}{{{{{{\rm{\rho }}}}}}}+\nabla \left({D}_{S}\nabla S\right)$$

Local sedimentation rates increase as a function of effective water layer thickness ${h}_{{{{{{\rm{e}}}}}}}$, i.e.

11 $${h}_{{{{{{\rm{e}}}}}}}=h-{H}_{{{{{{\rm{c}}}}}}}$$

which is a measure of inundation time, and asymptotically approach a spatially uniform, maximal value, ${S}_{{{{{{\rm{in}}}}}}}$. The rate at which the sedimentation rate increases with ${h}_{{{{{{\rm{e}}}}}}}$ is set by parameter ${Q}_{{{{{{\rm{s}}}}}}}$. Sediment erosion is dependent on the abiotic erodibility of sediment, ${E}_{S}$. The higher the sediment stabilizing potential ${p}_{{{{{{\rm{E}}}}}}}$ of vegetation is, the further vegetation can reduce this erodibility. As sediment erodibility generally decreases with depth due to consolidation^68^, erodibility is here assumed to be proportional to sediment elevation *S*. Finally, erosion is a function of bed shear stress ${\tau }_{{{{{{\rm{b}}}}}}}$. Inspired by earlier studies^12,32^, slope-driven sediment transport is modelled as a diffusive process. Sediment diffusivity ${D}_{S}$ is here assumed to be the susceptibility to slope-driven transport and inversely related to sediment cohesion. In line with previous studies^32^, the abiotic sediment diffusivity ${D}_{0}$ is assumed to be reduced by below-ground vegetative biomass, i.e.

12 $${D}_{S}={D}_{0}\left(1-{p}_{{{{{{\rm{D}}}}}}}\frac{B}{k}\right)$$

where the potential ${p}_{{{{{{\rm{D}}}}}}}$ of vegetation to reduce slope-driven transport can be seen as the extent of its root network. Finally, vegetation stem density *B* changes as a function of growth, mortality and lateral dispersion:

13 $$\frac{\partial B}{\partial t}={rB}\left(1-\frac{B}{k}\right)\left(\frac{{Q}_{{{{{{\rm{q}}}}}}}}{{Q}_{{{{{{\rm{q}}}}}}}+{h}_{{{{{{\rm{e}}}}}}}}\right)-{E}_{B}\, B\frac{{\tau }_{{{{{{\rm{b}}}}}}}}{\rho }+\nabla \left({D}_{B}\nabla B\right)$$

Following earlier studies^12,64^, logistic growth is assumed, with intrinsic growth rate *r*. Furthermore, vegetative growth is assumed to decrease asymptotically with local water depth (reaching half maximal growth rates when the effective water layer thickness ${h}_{{{{{{\rm{e}}}}}}}$ equals ${Q}_{{{{{{\rm{q}}}}}}}$), which is supported by experimental work showing that vegetation grows better on elevated micro-topography^41,48^. Biomass mortality is dependent on bed shear stress^50,51^, with ${E}_{B}$ the mortality rate per unit shear stress. Vegetative expansion is assumed to occur clonally only (via rhizomes) and is modelled as a diffusive process^18,39^, with lateral expansion rate (or diffusivity) ${D}_{B}$.

Initially, the topography is flat and horizontal, i.e., sedimentary elevation $S\left(t=0\right)=0$ everywhere. As a result, flow speeds are zero as well. A fixed initial water layer thickness, $h\left(t=0\right)={H}_{0}$ is imposed uniformly in the domain. Vegetation density is zero everywhere in the domain, apart from some sparse and randomly positioned tussocks. This initial vegetation distribution is determined by picking, for each grid cell, a random sample from a uniform distribution over [0,1]. Vegetation density of grid cells with random sample values < *p*_est_ (with ${p}_{{{{{{\rm{est}}}}}}}$ the probability of seedling establishment) are set to carrying capacity; the other cells remain unvegetated. The resulting heterogeneity in bed roughness, together with the bed slope that readily arises from the sedimentation-erosion balance, induces drainage flow and triggers the scale-dependent feedback.

The water that accumulates in the domain as a result of the water input term, ${H}_{{{{{{\rm{in}}}}}}}$, can drain out of the domain through the open boundary at $x={l}_{x}$ (Supplementary Fig. 8). At this boundary, which represents the connection of the intertidal area with the main tidal channel, it is assumed that there is a constant gradient in flow speed, i.e., $\frac{{\partial }^{2}u}{\partial {x}^{2}}=0$ and $\frac{{\partial }^{2}v}{\partial {x}^{2}}=0$. At this boundary, there is furthermore assumed to be no gradient in water layer thickness and vegetation. Erosion and deposition are assumed to be in balance along this boundary, such that sediment elevation remains zero. The other three boundaries are assumed to be closed, i.e., water flow reflects off these boundaries and there is no gradient in water layer thickness, sediment elevation and vegetation density at these three boundaries.

### Numerical implementation

Equations (3)–(10), (12) and (13) are solved numerically, using forward Euler discretization in time and central differencing in space. The time step size is $\Delta {{{{{\rm{t}}}}}}$ and the spatial grid coordinates are at $x=i\,\Delta {{{{{\rm{x}}}}}}$, with $i=\left({{{{\mathrm{0,1,2}}}}},\ldots,{n}_{x}-1\right)$ and ${l}_{x}={n}_{x}\Delta {{{{{\rm{x}}}}}}$, and idem $y=j\,\Delta {{{{{\rm{y}}}}}}$. All equations can be discretized in a straightforward manner, but the topographic diffusion term in Eq. (10), i.e., $\nabla \left({D}_{S}\nabla S\right)$, requires extra attention. This term is discretized as follows:

14 $${\left(\frac{\partial }{\partial x}\left({D}_{S}\frac{\partial S}{\partial x}\right)\right)}_{i,j}=\frac{1}{\Delta {{{{{\rm{x}}}}}}}\left({\left({D}_{S}\frac{\partial S}{\partial x}\right)}_{i+\frac{1}{2},j}-{\left({D}_{S}\frac{\partial S}{\partial x}\right)}_{i-\frac{1}{2},j}\right)$$

and idem in the *y*-direction. Here, e.g.,

15 $${\left({D}_{S}\frac{\partial S}{\partial x}\right)}_{i+\frac{1}{2},j}={\left({D}_{S}\right)}_{i+\frac{1}{2},j}\frac{{S}_{i+1,j}-{S}_{i,j}}{\Delta {{{{{\rm{x}}}}}}}$$

and ${\left({D}_{S}\right)}_{i+\frac{1}{2},j}$ is calculated by linearly interpolating between ${\left({D}_{S}\right)}_{i,j}$ and ${\left({D}_{S}\right)}_{i+1,j}$.

At the open outflow boundary, the constant gradient in *u* is discretized as

16 $$u\left(i={n}_{x}-1,j\right)=2u\left({n}_{x}-2,j\right)-u\left({n}_{x}-3,j\right)$$

and identically for *v*. Zero gradient at this boundary is discretized as

17 $$h\left(i={n}_{x}-1,j\right)=h\left({n}_{x}-2,j\right)$$

for water depth *h*, and idem for vegetation density *B*. At the closed boundary opposite the outflow boundary, i.e., at $x=0$, the assumption of reflecting water flow is discretized as

18 $$u\left(i=0,j\right)=-u\left(i=1,j\right)$$

19 $$v\left(i=0,j\right)=+ v\left(i=1,j\right)$$

and similarly, but with reversed plus and minus signs, for the closed boundaries at $y=0$ and $y={l}_{y}$. At every closed boundary, the zero gradient conditions for *h*, *S* and *B* are implemented similarly as above for $h\left(i={n}_{x}-1,j\right)$.

At the beginning of each time step, it is first ensured that the water layer *h* is nowhere shallower than the critical value, as in Eq. (4). After that, Eqs. (5) and (6) are calculated (where all variables still have the values from the old time step), followed by an update of *u* and *v*, i.e.

20 $$u\left(t+\Delta {{{{{\rm{t}}}}}}\right)=u\left(t\right)+\frac{\partial u}{\partial t}\Delta {{{{{\rm{t}}}}}}$$

and idem for *v*. Boundary conditions on *u* and *v* are imposed hereafter. Then, $\frac{\partial h}{\partial t}$, $\frac{\partial S}{\partial t}$ and $\frac{\partial B}{\partial t}$ are computed (with updated values for *u* and *v*) and *h*, *S* and *B* are updated, followed by enforcement of the boundary conditions.

Since sedimentary and vegetative dynamics, i.e., Eqs. (10) and (13), are inherently slower than hydrodynamics, i.e., Eqs. (3), (5) and (6), the two biogeomorphic equations are here numerically accelerated, using a morphological acceleration factor, which is a well-established method in biogeomorphic modelling studies^12,29^, Φ, i.e.

21 $$S\left(t+\Delta {{{{{\rm{t}}}}}}\right)=S\left(t\right)+\frac{\partial S}{\partial t}\Delta {{{{{\rm{t}}}}}}\, \Phi$$

and idem for *B*. Thanks to the simplified sedimentary Eq. (10), wherein the advective terms have been neglected, morphological acceleration does not lead to numerical instabilities due to a violation of the CFL condition. Throughout this study, whenever we mention a simulated time, we refer to the morphologically accelerated time, i.e., in terms of biogeomorphic processes. The exact parameter settings of all simulations, performed with the numerical model, can be found in Tables 1–3. To allow high-speed calculations, the numerical model is implemented on graphics processing units (GPU), as is further described hereafter.

The numerical model is programmed in Python (https://www.python.org/). The entire code is implemented in a Jupyter Notebook. The computing kernel itself, however, is written in OpenCL, using the Python package PyOpenCL. This allows the code to directly access the graphics processing units (GPU) or central processing unit (CPU) of the computer that is being used. GPU’s can handle large numbers of operations in parallel, on multiple threads^69^, which enables a drastic speed-up and/or scale-up of the numerical model. The model simulations are performed on a Tesla P100-PCIE-12GB GPU, but can in principle be performed on any device with sufficiently powerful GPU or CPU. As a consequence of this model implementation, the discretized numerical calculations are not handled as matrix-operations. Instead, the spatial grid is subdivided into a large number of thread blocks, each containing a number of threads (each accounting for one grid cell). These thread blocks run individually from each other but exchange information (e.g., exchanges between thread block areas) every fixed number of timesteps. Although this might in theory result in asynchrony between thread block operations, we found that the model results are not negatively affected by this parallelization.

To study how abiotic topographic diffusivity (${D}_{0}$) affects the simulated channel pattern, a gradient simulation was performed (Fig. 3a). The simulated domain was extended further in the *y*-direction and ${D}_{0}$ was varied along this coordinate. To cover several orders of magnitude for ${D}_{0}$, the parameter was varied between ${D}_{0,\min }$ and ${D}_{0,\max }$, following

22 $${D}_{0}\left(y\right)=\exp \left(\left({{{{\mathrm{ln}}}}}[{D}_{0,\max }]-{{{{\mathrm{ln}}}}}[{D}_{0,\min }]\right)\frac{y}{{l}_{y}}+{{{{\mathrm{ln}}}}}[{D}_{0,\min }]\right)$$

However, the analyses in Fig. 3b are not performed directly on the simulation in Fig. 3a. Instead, this gradient simulation is split up into normal simulations (i.e., with rectangular domain size, similar to Fig. 1f). The value of ${D}_{0}$ varies between these separate simulations, following the gradient in Fig. 3a, but is constant within each individual simulation. This is done because gradient simulations visualize well the qualitative effect of a parameter, but since adjacent areas along this gradient can interfere with each other, it is better to separate these areas for the purpose of a more quantitative analysis.

### Derivation of model parameters

Model parameters whose values could not be directly obtained from the literature can be divided into two categories: empirically tuned parameters and derived parameters, denoted as e and d, respectively, in Table 2. The derivation of the latter set of parameters is explained here.

The tidally averaged discharge velocity, ${H}_{{{{{{\rm{in}}}}}}}$, was calculated by assuming that the entire volume of water, elevated above the lower boundary of the modelled tidal flat area, drains away within one tidal period. In an earlier field study, algal mat-induced drainage patterns were observed on a tidal flat that sloped upwards, starting from about 1.5 m below MHWS^19^. Assuming that the tidal flat slope is uniform within this elevation range, this translates into a discharge (calculated as the drained volume per unit tidal flat area during spring tide) of 0.75 m per tidal period T_M2_, or about 1.7*10^−5^ m s^−1^. This gives an order-of-magnitude estimate; after some sensitivity analyses, we found that the (visually) most realistic channel patterns were obtained at *H*_in_ = 1.0*10^−5^ m s^−1^.

As a consequence of the steady discharge-assumption mentioned above, the larger water depths encountered during a real tidal cycle are not simulated in our model. As a result, the amount of sediment contained in these shallower water depths is also smaller than under real (larger water depth) conditions. We start from the maximal sediment input value of $\bar{{S}_{{{{{{\rm{in}}}}}}}}=0.2{{{{{\rm{cm}}}}}}{[{{{{{{\rm{T}}}}}}}_{{{{{{\rm{M}}}}}}2}]}^{-1}$, as used in earlier model studies^12^, and reduce this value by a factor of 10, to account for the smaller water depths in our model. In our approach, the effective sediment input is also a function of the actual water depth, i.e. $\bar{{S}_{{{{{{\rm{in}}}}}}}}$ in the previous study^12^ is equivalent to ${S}_{{{{{{\rm{in}}}}}}}\frac{{h}_{{{{{{\rm{e}}}}}}}}{{Q}_{{{{{{\rm{s}}}}}}}+{h}_{{{{{{\rm{e}}}}}}}}$ in our study. Assuming $h={H}_{0}$ (hence ${h}_{{{{{{\rm{e}}}}}}}={H}_{0}-{H}_{{{{{{\rm{c}}}}}}}$), and having reduced $\bar{{S}_{{{{{{\rm{in}}}}}}}}$ with a factor 10 relative to the earlier model study^12^, we obtain ${S}_{{{{{{\rm{in}}}}}}}\approx 5*{10}^{-9}{{{{{\rm{m}}}}}}{{{{{{\rm{s}}}}}}}^{-1}$.

In line with the derivation of ${S}_{{{{{{\rm{in}}}}}}}$ (explained above) and in order to maintain a sedimentation-erosion balance, the erosion rate $\bar{{E}_{S}}$ is also reduced by a factor of 10, compared to the earlier model study^12^, i.e. $\bar{{E}_{S}}=(0.03{{{{{{\rm{tide}}}}}}}^{-1})/10=0.003{{{{{{\rm{tide}}}}}}}^{-1}$. In our model, sediment erosion rate is also a function of bed shear stress, i.e. $\bar{{E}_{S}} \sim {E}_{S}\frac{{\tau }_{{{{{{\rm{b}}}}}}}}{\rho }$. Under homogeneous equilibrium, $\frac{{\tau }_{{{{{{\rm{b}}}}}}}}{\rho }$ can be obtained from the momentum Eq. (5), i.e. this equation reduces to a balance between pressure gradient force and bed shear stress: $0=g\beta -\frac{{\tau }_{{{{{{\rm{b}}}}}}x}}{\rho h}$, where the pressure gradient term has been computed under the assumption of a uniform sub-sediment bed slope on the order of 10^−3 ^m m^−1^. Taking $h={H}_{0}$ and $v=0$, and using the ten-fold reduced value of $\bar{{E}_{S}}$, this yields an order-of-magnitude estimate for ${E}_{S}$. After some sensitivity analyses, we yield the visually most realistic results for ${E}_{S}=2.5*{10}^{-4}{{{{{\rm{s}}}}}}{{{{{{\rm{m}}}}}}}^{-2}$.

### Extraction of real-world and simulated channel networks

To extract tidal network properties, the digital terrain models (DTMs) of simulated marshes (i.e., sediment elevation matrices) and the DTMs of four real-world tidal marshes were used. The DTM of Saeftinghe (dating from 2014; located around 51°22'11.47“N, 4°11'14.18“E) was acquired from the Actueel Hoogtebestand Nederland (AHN3, openly accessible under CC0 1.0 Universal license from https://app.pdok.nl/ahn3-downloadpage/). The DTMs of Leysdown-on-Sea (dating from 2020; located around 51°21′45.44“N, 0°52'12.48“E), Humber East (2017; 53°40'59.56“N, 0°11'13.77“W) and Humber West (2017; 53°42'2.43“N, 0°12'49.35“W) were collected by the Environment Agency (EA) and acquired under the Open Government Licence v3.0 (© Crown Copyright 2022) from their data service platform (Defra Data Services Platform, https://environment.data.gov.uk/DefraDataDownload/?Mode=survey). All DTMs have a 0.5 m x 0.5 m pixel size and vertical accuracy of 0.1 m (Saeftinghe) and 0.15 m (the three UK marshes). The tidal marshes range from well-developed (Saeftinghe and Leysdown-on-Sea) to newly developed (Humber East) to poorly developed (Humber West). This is deduced from historical cartography (source: Kadaster, https://www.topotijdreis.nl/) which indicates that the Saeftinghe marsh formed between 1949 and 1960, and aerial images (source: Google Earth Pro) which suggest that the marsh at Leysdown-on-Sea formed between 1940 and 1960 or 1985, the Humber East marsh between 2007 and 2017, and the Humber West marsh between 2017 and 2021.

Channel networks and their properties were extracted from these simulated and real-world DTMs with the Python package TidalGeoPro^70,71^ (version 0.4 - 10.5281/zenodo.7071308). This package first uses median neighborhood analysis to identify channel pixels. The channel contours (polygons) are then determined and unchanneled path lengths are calculated as the Euclidian distance to the closest channel pixel. The stream skeletons are determined as the centerlines of the channel polygons and redefined at equidistant (0.5 m) points along the entire skeleton. At each skeleton point and each skeleton node, the maximum and total upstream length and watershed surface area are computed. For each skeleton section (connection between two skeleton nodes), the stream order is computed following Hack’s definition.

The output of the analysis with the TidalGeoPro package is used for further analysis. Each skeleton point is assigned to the subbasin where it belongs to. For each main stream (Hack stream order 1), the most upstream node (channel head) is identified. Each 1st-order stream is then followed downstream until the downstream-most node (i.e., where the channel reaches the outflow boundary). A unique identifier is assigned to each of these channels, to classify them as the main channel of their respective 1st-order subbasin. This procedure is repeated, now following each 2^nd^-order channel head downstream until its confluence with a 1st-order channel, hence identifying the individual 2^nd^-order channels that constitute the main channels of 2^nd^-order subbasins, and so on for increasingly high Hack stream order. The number of individual channels (or subbasins), *n*_*i*_, of each Hack stream order *i* are counted to compute bifurcation ratios, *r*_*i*_, as *r*_*i*_ = *n*_*i+1*_/*n*_*i*_ (Fig. 4c). For the analyses in Fig. 4a and Supplementary Fig. 7a, the watershed surface area data computed at each equidistant skeleton point were used. Analyses in Fig. 4b and Supplementary Fig. 7b use the unchanneled path lengths, calculated at each unchanneled grid cell. Hortonian drainage densities (Fig. 3b and Supplementary Fig. 7c) are computed as the total network length divided by the total watershed surface area of a given tidal marsh^41^.

The simulated and real-world channel network properties were quantitatively compared by calculating the two-sample Kolmogorov-Smirnov test for goodness of fit. The probability distributions of watershed surface area (calculated at each equidistant point along the stream skeleton) of each simulated and real-world network were compared in Python (scipy.stats.ks_2samp) and (1-KS), with KS the KS-statistic, was used as measure of similarity. The same approach was taken for the unchanneled path lengths (calculated for each unchanneled grid cell). For all comparisons between real-world and simulated data, the null hypothesis of the KS-test (i.e., the hypothesis that real-world and simulated data were drawn from the same distribution) was rejected (p-value equal to zero), because the distributions were always significantly different from each other. However, the trends in the KS-values can still be used to determine how the strength of the scale-dependent feedback (i.e., the value of ${D}_{0}$) affects the relative similarity between real-world and simulated networks.

### Observations of nested channels on Ketenisse tidal flat

The observations shown in Supplementary Fig. 9a–c are done on the Ketenisse intertidal flat, located in the Scheldt estuary in Belgium, close to the Dutch border (around 51°17'4.09“N, 4°18'45.44“E). This intertidal flat consists of formerly embanked agricultural land, in which tidal influence has been reintroduced in 2003 by managed realignment of the enclosing dikes. From aerial images obtained with Google Earth Pro, © 2022 Google LLC, it can be deduced that on 8 June 2004, i.e., shortly after de-embankment of this area, a tidal channel is incised in the newly deposited sediments (Supplementary Fig. 9a). A few years later, clear second-order side channels have emerged (Supplementary Fig. 9b). These second-order channels have a regular spacing of about 2–3 m.

As reported in earlier work^19^, a large part of this intertidal flat became colonized by biofilms of the algae *Vaucheria sp*. From Google Earth aerial images it can be inferred that algal cover also extended to the banks of this drainage channel. Aerial imagery and in-situ observations^19^ show that these algal biofilms enhance the self-organization of single-scaled, regularly spaced ridge-runnel bedforms. The pattern of algal-covered elevated ridges alternating with bare low-lying runnels can also be seen in aerial images of the large tidal channel (Supplementary Fig. 9b). From this, together with in-situ observations that this regularly-spaced and meter-scale alternation of ridges and runnels is, by eye, very similar to the ridge-runnel pattern reported earlier^19^, we infer that these second-order side-channels are most likely formed by the same scale-dependent feedback mechanism.

In-situ observations in 2016 furthermore reveal that ripple-like third-order channels are incised on the banks of these second-order channels (Supplementary Fig. 9c). These third-order channels also seem to have a regular spacing, on the order of 10 cm. Although it cannot be said with complete certainty, because in-situ observations from before 2015 are not available, it seems most likely that these third-order patterns have formed after the emergence of the second-order channels. Moreover, although these channels somewhat resemble wave ripples as well, it seems most likely that these are drainage runnels instead. This is suggested by the observation that the third-order channels are oriented down the bank slopes of the second-order channels, irrespective of the orientation of these second-order channels, rather than towards the estuary where waves would originate.

We hence conclude that these observations showcase a sequence of nesting of regular patterns, to form a complex, multi-scale regular pattern. Together with the earlier finding that single-scaled patterns on the same tidal flat have self-organized due to biogeomorphic scale-dependent feedbacks^19^, we conclude that this is a real-world example of the recursive biogeomorphic feedback mechanism that we have analyzed in the current study. The fact that these observations were done in a tidal channel network colonized by biofilms rather than plants, further supports the idea that, apart from enhanced channel incision due to vegetative roughness (plant stems), an algal-induced increase in soil strength (hence a reduction of topographic diffusion) can equally lead to the formation of higher-complexity channel patterns.

### Supplementary information

Supplementary Information

## Data Availability

The data that supports the findings of this study are archived and publicly available via 10.4121/8d361887-ec02-4472-a8eb-a9d0f3eacfd6. Source data are provided for Figs. 1–4 and Supplementary Figs. 1–7, and are available as a Source Data file via 10.4121/8d361887-ec02-4472-a8eb-a9d0f3eacfd6.

## Code availability

The codes that support the findings of this study are archived and publicly available via: 10.4121/8d361887-ec02-4472-a8eb-a9d0f3eacfd6. The SFERE (Scale-dependent Feedback Recursion) model (v1.0) that was introduced in this study is available via GitHub (https://github.com/RCvandeVijsel/SFERE) and Zenodo (10.5281/zenodo.8428792).
