# Supplementary material for: Vegetation controls on channel network complexity in coastal wetlands
Source: Nat Commun. 2023 Nov 7;14:7158. doi: 10.1038/s41467-023-42731-3 (PMC10630343; doi:10.1038/s41467-023-42731-3)
Supplement: Supplementary file 1 — Supplementary Information [file 41467_2023_42731_MOESM1_ESM.pdf]

Supplementary Information for:

Vegetation controls on channel network complexity in coastal wetlands

Nature Communications (2023)

Roeland C. van de Vijsel[1,2,3*], Jim van Belzen[1,4,5], Tjeerd J. Bouma[1,2,6], Daphne van der Wal[1,7], Bas W. Borsje[8], Stijn Temmerman[5], Loreta Cornacchia[1,9], Olivier Gourgue[5,10], Johan van de Koppel[1,2]

[1] Department of Estuarine and Delta Systems, NIOZ Royal Netherlands Institute for Sea Research, Yerseke, The Netherlands

[2] Groningen Institute for Evolutionary Life Sciences, University of Groningen, Groningen, The Netherlands

[3] Now at: Hydrology and Environmental Hydraulics Group, Wageningen University, Wageningen, The Netherlands

[4] Wageningen Marine Research, Wageningen University and Research, Yerseke, The Netherlands

[5] Ecosphere Research Group, University of Antwerp, Antwerp, Belgium

[6] Department of Physical Geography, Faculty of Geosciences, Utrecht University, Utrecht, The Netherlands

[7] Faculty of Geo-Information Science and Earth Observation, University of Twente, Enschede, The Netherlands

[8] Water Engineering and Management, University of Twente, Enschede, The Netherlands

[9] Now at: Marine and Coastal Systems, Deltares, Delft, The Netherlands

[10] Operational Directorate Natural Environment, Royal Belgian Institute of Natural Sciences, Brussels, Belgium

*e-mail: roeland.vandevijsel@wur.nl

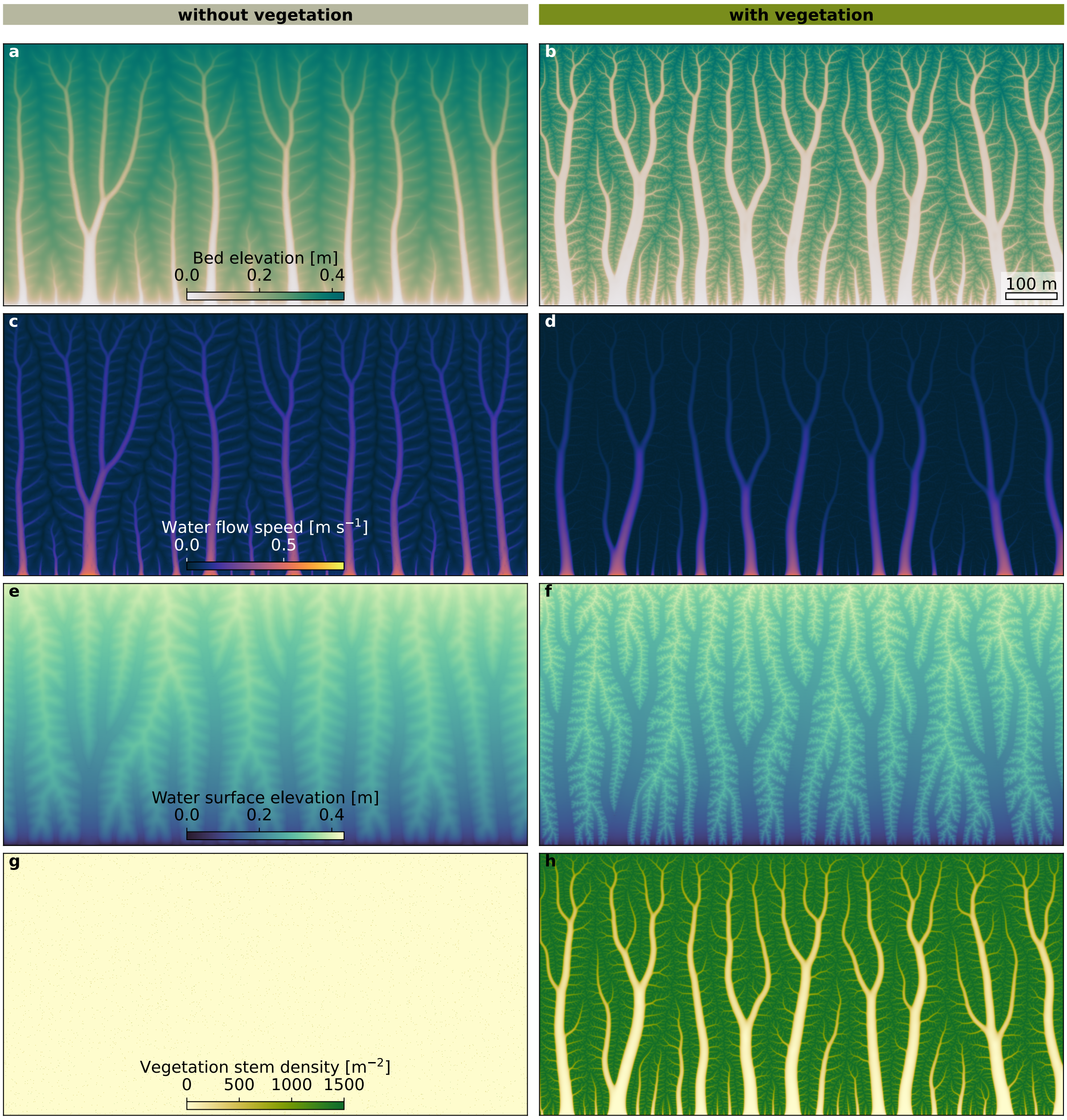

Supplementary Fig. 1 | Overview of all variables in the default tidal network simulations of Fig. 1e,f in the main manuscript, in the absence (left column) and presence (right column) of vegetation. **a**,**b**, Sediment bed elevation S. **c**,**d**, Absolute depth-averaged flow speed $\sqrt{u^2 + v^2}$. **e**,**f**, Water surface elevation $\eta = S + h$ with water layer thickness h. **g**,**h**, Vegetation density B. Simulations after 50 simulated years. The bottom boundary is an open outflow boundary, the other three boundaries are closed. Source data are provided as a Source Data file.

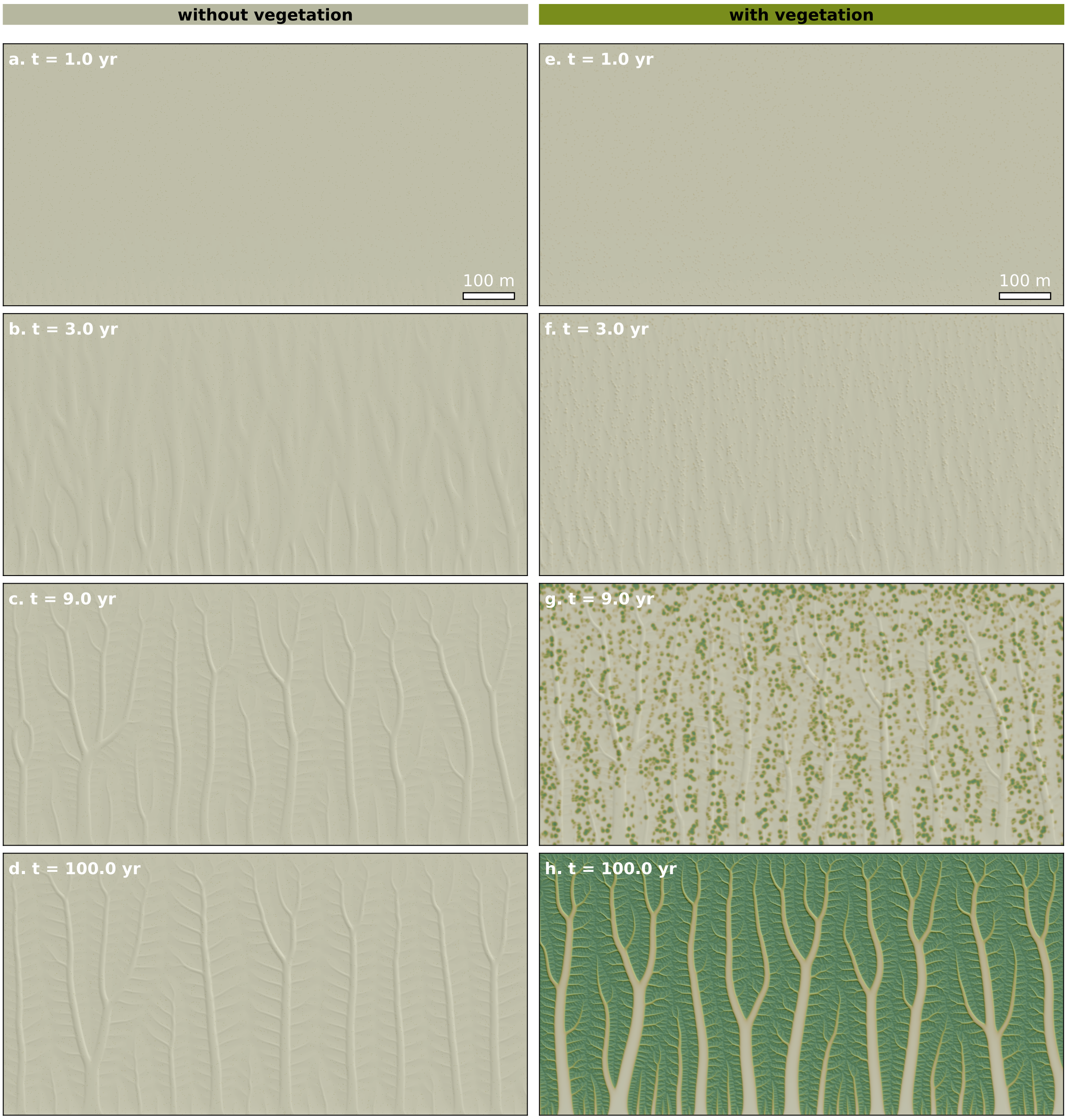

Supplementary Fig. 2 | Temporal development of simulated tidal network, in the absence (left column) and presence (right column) of vegetation. The maps show vegetation density in green colors, with topograhy (shaded relief, in grey colors) as a semi-transparent overlay. The exact values of topography and vegetation density are given in Supplementary Fig. 1. **a**,**e**, Initially, bathymetry is flat, with sparse and heterogeneous vegetation cover (small pixels, thus hardly visible). **b**,**f**, After some time, when the bed starts sloping due to local sedimentation, scale-dependent feedbacks create a fine-scaled and braided channel pattern that propagates upslope and is finer-scaled in the vegetated case. **c**,**g**, The channel network erodes further onto the vegetated wetland platform and fine braiding streams merge into fewer, deeper channels. **d**,**h**, The channel network becomes increasingly complex over time, developing higher-order side-channels. Vegetated network reach significantly higher channel branching orders than unvegetated networks. When the simulation is continued longer, the channels become straighter and the nested channel pattern more regular. All settings as in default model runs Fig. 1f in the main manuscript and Supplementary Fig. 1. Source data are provided as a Source Data file.

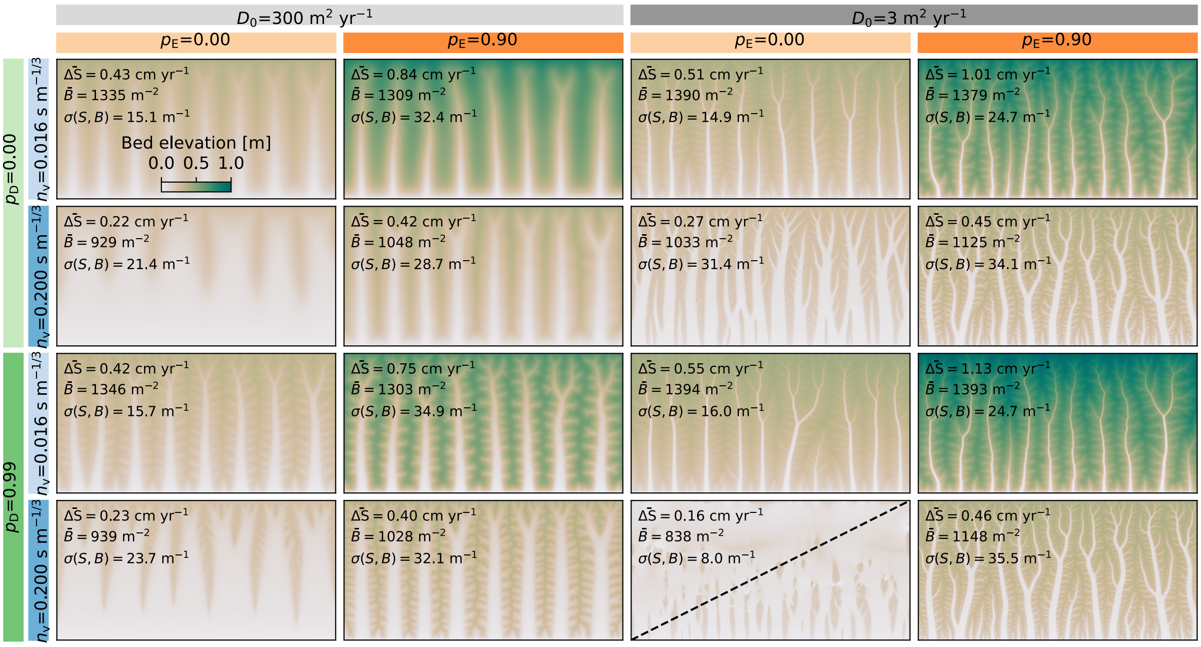

Supplementary Fig. 3 | Effect of different vegetation parameters on channel network morphology. The maps show sediment bed elevation after 50 years of simulation. Rows marked with blue panels show the effects of vegetation-induced bed roughness (Manning's coefficient for vegetated beds, n_v), i.e. simulations without vegetative roughness (light-blue rows, i.e. n_v equals bare-sediment roughness n_b) or with such an effect (dark blue rows). Columns marked by orange panels show the effect of vegetation-induced erosion-protection (the factor by which vegetation decreases abiotic soil erodibility, p_E), i.e. simulations without vegetation-induced erosion protection (light orange columns) and with such an effect (dark orange columns). Rows marked by green panels show the effect of vegetation-induced soil strengthening (the factor by which vegetation reduces soil diffusion, p_D), i.e. simulations without vegetation-induced soil strengthening (light green rows) and with such an effect (dark green rows). The left half of the simulations (i.e., columns indicated by light gray bars) are simulations with relatively high abiotic soil diffusivity ($D_0 = 300$ m² yr⁻¹), whereas the right half of the simulations (i.e., columns indicated by dark gray bars) are simulations with relatively low abiotic soil diffusivity ($D_0 = 3$ m² yr⁻¹). The panel with a diagonal dashed line showed some numerical instabilities during simulation which could not be resolved in a straightforward manner, but is shown here for completeness. The values in each subplot indicate average accretion rates, $\overline{\Delta S}$ (averaged over the spatial domain and over the simulation time), domain-averaged vegetation densities, $\bar{B}$, and the covariance of sediment elevation and vegetation density, $\sigma(S, B)$. See Table 3 in the main manuscript for complete parameter settings. Source data are provided as a Source Data file.

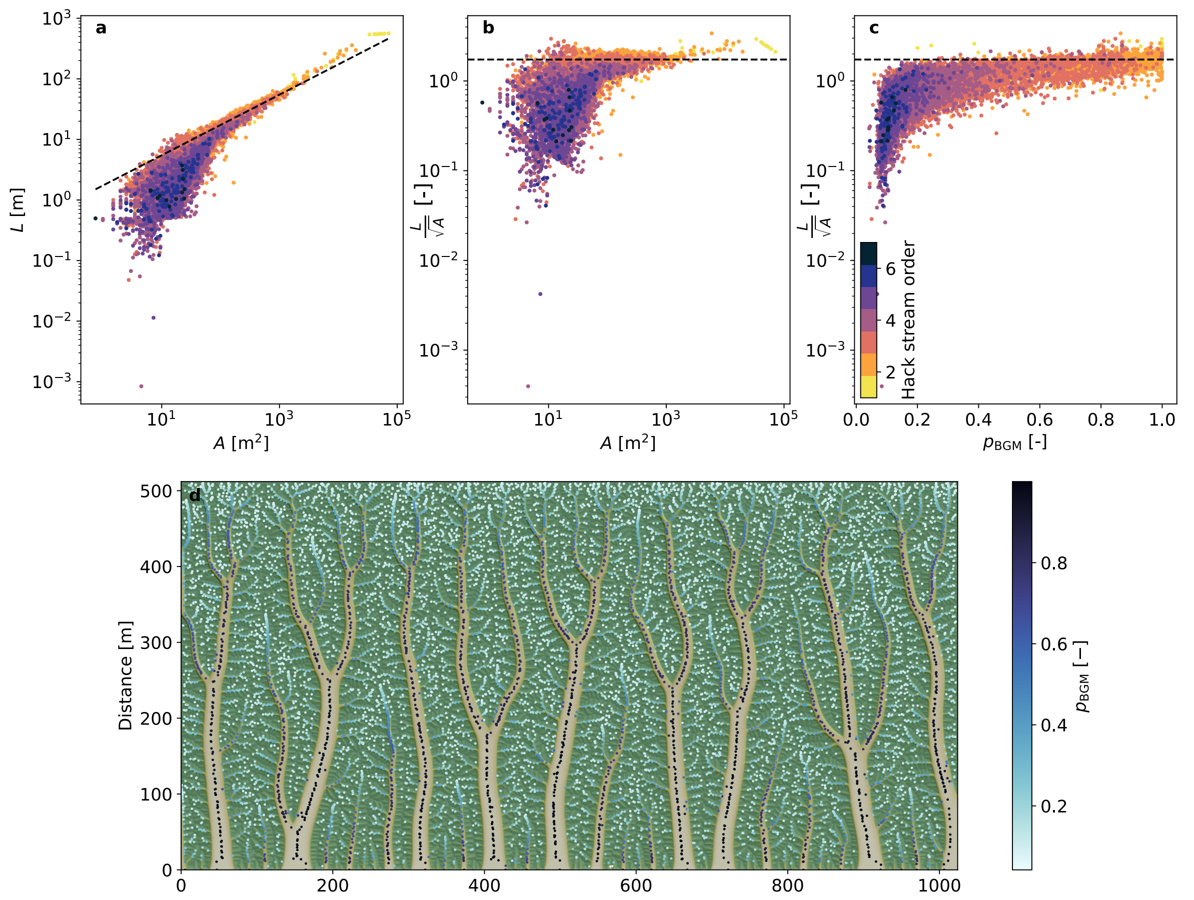

Supplementary Fig. 4 I Network refinement depends on the potential of vegetation to induce the scale-dependent biogeomorphic feedback. a, The scaling relation between watershed drainage area, A, and the maximum upstream channel length, L, follows the theoretical[53] power law $L = \sqrt{3A}$ (black dashed line) for relatively large subbasins, but starts to break off from this scaling relation for smaller subbasins, indicating that for a given subbasin area, the incised channels are shorter than expected from the theoretical relationship. Colors indicate Hack stream order of the respective subbasin. **b**, Idem, but now the y-axis shows $L/\sqrt{A}$ instead. **c**, For each subbasin, the potential of vegetation to induce a scale-dependent biogeomorphic feedback, here called p_{BGM}, is plotted. This number equals the normalized difference between the vegetation carrying capacity and the actual vegetation density in the channel at the downstream origin of a given subbasin, i.e., $p_{\mathrm{BGM}} = \frac{k-B_c}{k}$, where k is the carrying capacity and B_c is the vegetation density in the middle of the channel at the most downstream location of each subbasin. When $p_{\mathrm{BGM}} = 0$, vegetation can fully colonize the channel and the scale-dependent nature of the biogeomorphic feedback is lost, while this scale-dependent feedback is active when p_{BGM} is larger than 0 (and maximally 1). This plot shows that, once the biogeomorphic feedback starts to break down (i.e., p_{BGM} decreases), the incision of channels becomes less efficient, as measured by the increasing offset of $L/\sqrt{A}$ from the theoretically expected value of $\sqrt{3}$. **d**, The quantity p_{BGM} is plotted at each downstream origin of the subbasin where it was calculated, and shown within the simulated marsh network (vegetation density indicated in green colors; bed elevation indicated as shaded relief map in grey colors). The map shows how p_{BGM} eventually decreases as channels get exceedingly fine-scaled, indicating that vegetation reaches the limit of the scale at which it still is affected by (the now very subtle) topographic and hydrodynamic gradients. See Table 3 in the main manuscript for model parameter settings. Source data are provided as a Source Data file.

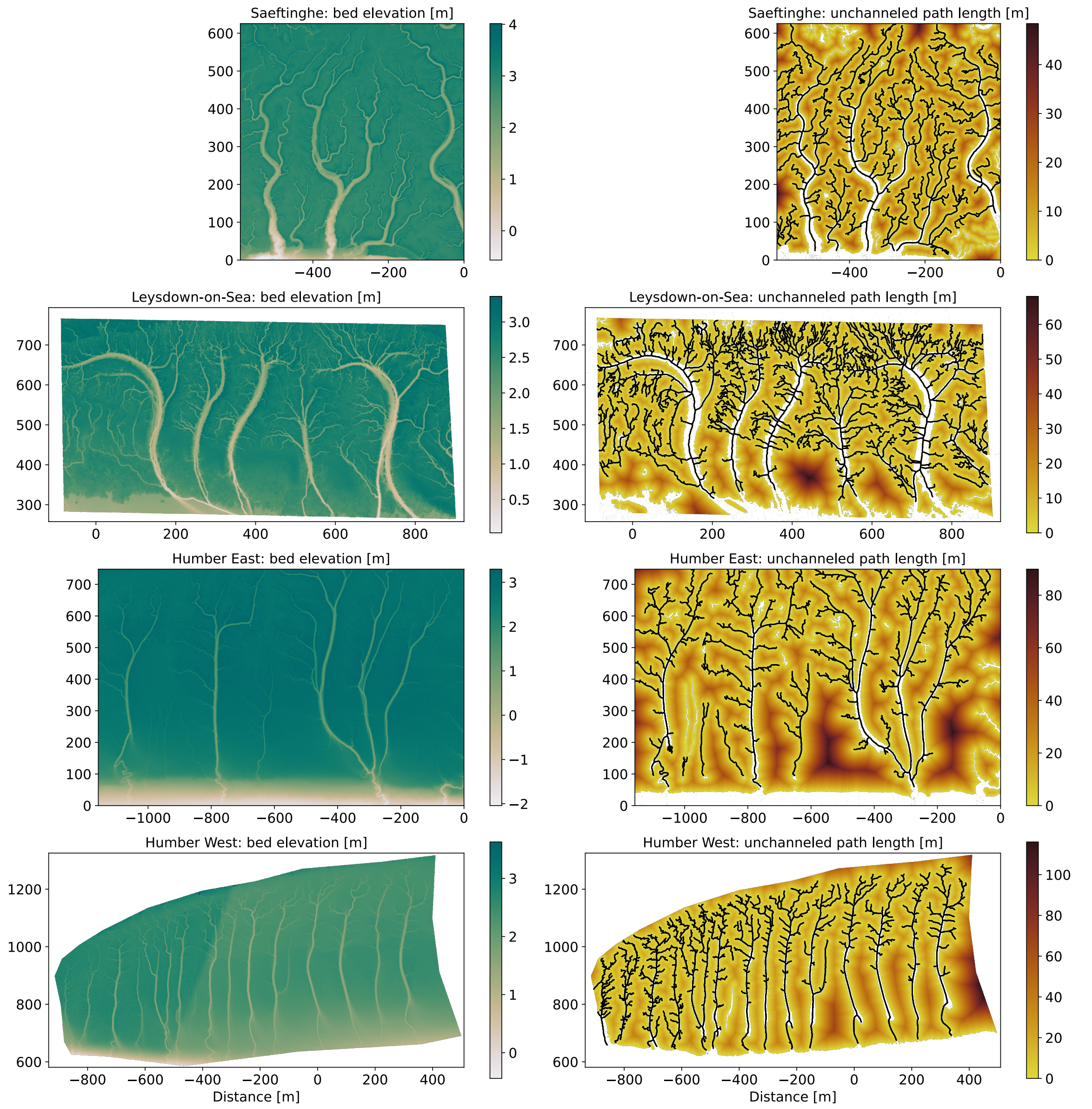

Supplementary Fig. 5 I DTMs (left column) and unchanneled path lengths with stream skeletons (right column) of the four real-world tidal marshes. The DTM of Saeftinghe was acquired from the Actueel Hoogtebestand Nederland (AHN3, openly accessible under CC0 1.0 Universal license from https://app.pdok.nl/ahn3-downloadpage/). The DTMs of Leysdown-on-Sea, Humber East and Humber West were collected by the Environment Agency (EA) and acquired under the Open Government Licence v3.0 (© Crown Copyright 2022) from their data service platform (Defra Data Services Platform, https://environment.data.gov.uk/DefraDataDownload/?Mode=survey). Source data are provided as a Source Data file.

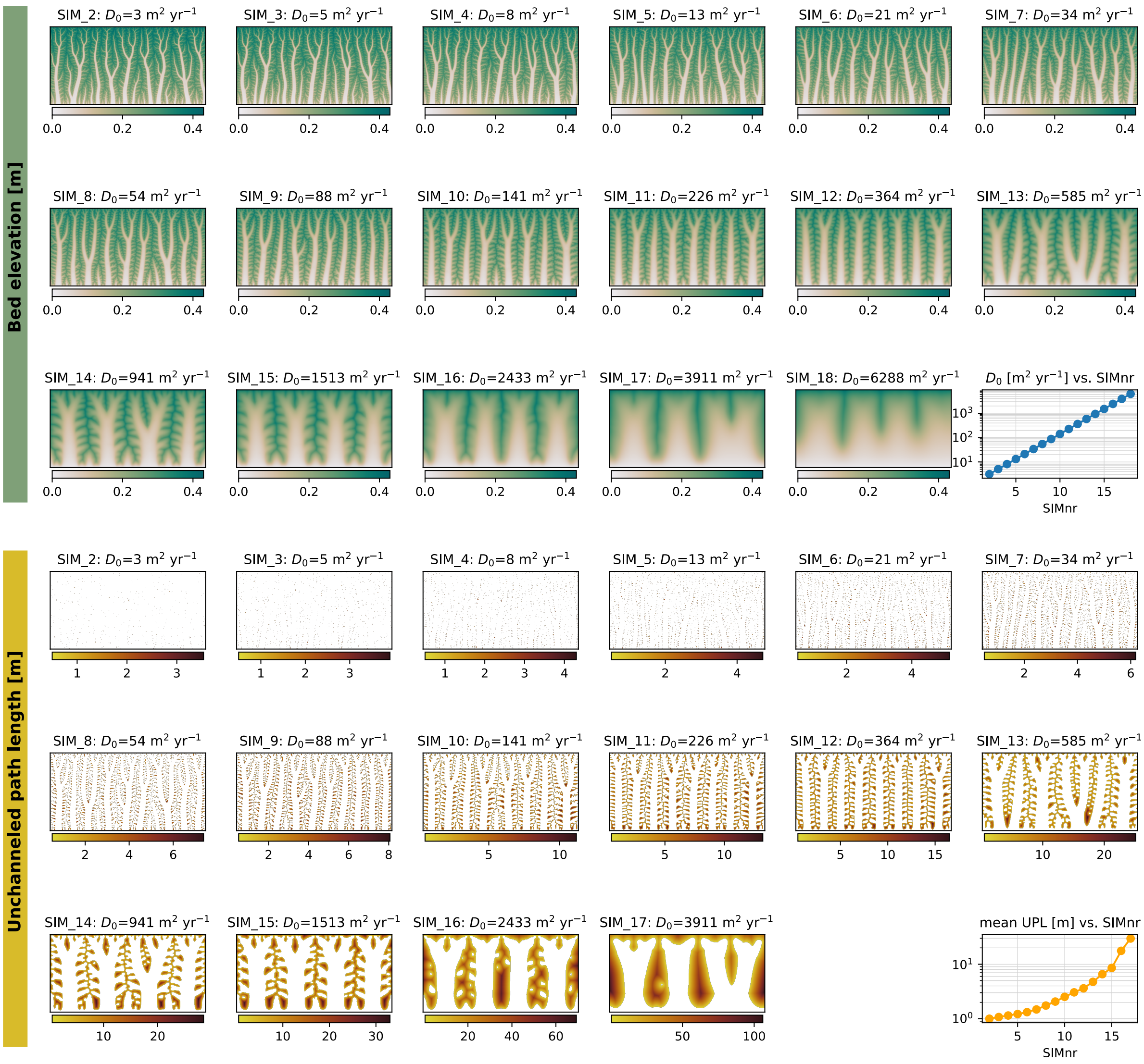

Supplementary Fig. 6 | Simulated DTMs (sediment bed elevation) and corresponding unchanneled path lengths for the 17 simulations along the D_0-gradient in Fig. 3 in the main manuscript. Top half (bed elevation): due to the extremely wide and smooth bathymetry of SIM_18 (i.e., the simulation with the highest value of D_0), the channel network could not be extracted for this simulation. For this reason, only SIM_2 until SIM_17 are shown in Fig. 3b. The bottom-right panel of the DTMs shows the value of D_0 as a function of the simulation number. Bottom half (unchanneled path lengths): unchanneled path lengths for the 16 simulations along the D_0-gradient in Fig. 3. White pixels indicate channelized areas. Please note that SIM_18 is not shown, since the channel network could not be extracted for this extremely wide and smooth network. The lower right panel indicates the mean value of all unchanneled pixels, for each simulation. Source data are provided as a Source Data file.

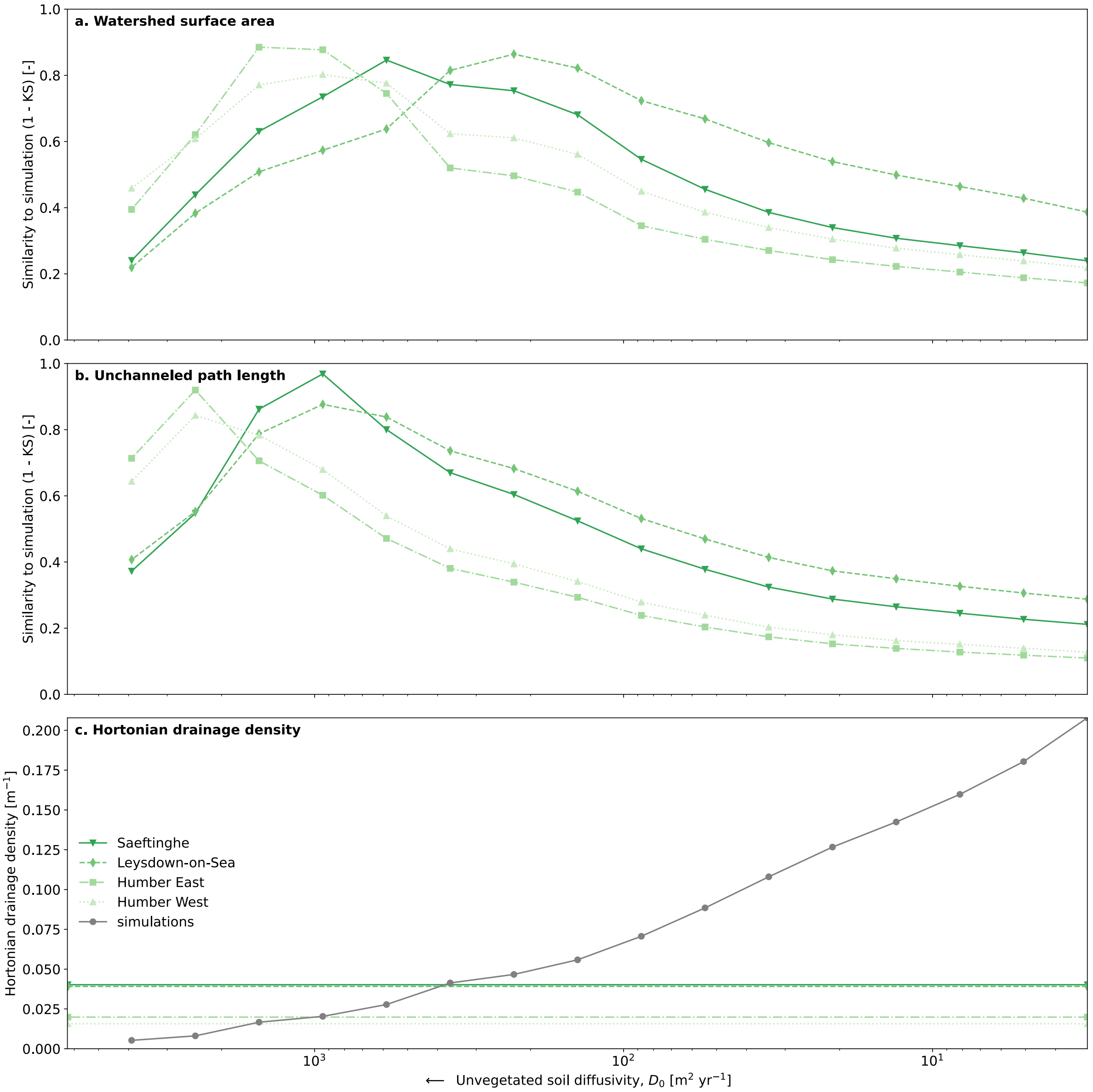

Supplementary Fig. 7 | Network characteristics of the four real-world channel networks, compared to the simulated networks along the D_0-gradient in Fig. 3 in the main manuscript. a, Comparison of the probability distributions of watershed surface areas. The similarity between real-world and simulated networks is expressed as (1-KS), where KS is the KS statistic of the two-sample Kolmogorov-Smirnov test for goodness of fit, i.e., 1-KS=0 means perfect disagreement and 1-KS=1 means perfect agreement. **b**, Idem, but for the probability distributions of unchanneled path lengths. **c**, Hortonian drainage densities, computed for real-world networks (horizontal lines) and simulated networks (grey data points). Note that network geometries of the maximal D_0-value along the gradient could not be calculated, because the digital terrain model was too smooth. Source data are provided as a Source Data file.

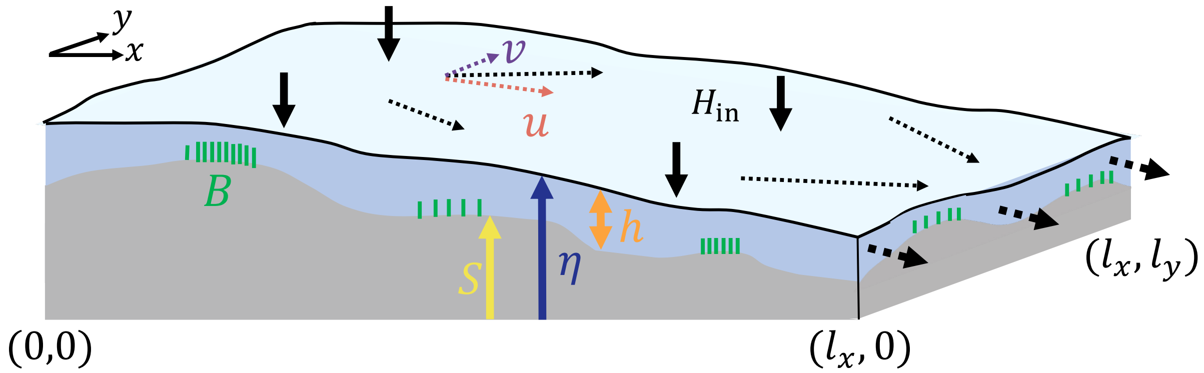

Supplementary Fig. 8 | Schematic representation of the model setup. The downslope and cross-slope coordinate are given by x and y respectively, and their corresponding depth-averaged water flow components are u and v. The water layer thickness is given by h, total water surface elevation by η, sediment bed elevation by S and vegetation stem density by B. The continuous water input rate (tidally averaged discharge velocity) is given by H_{in}. All lateral boundaries ($x = 0$, $y = 0$ and $y = l_y$) are closed, except the open outflow boundary at $x = l_x$.

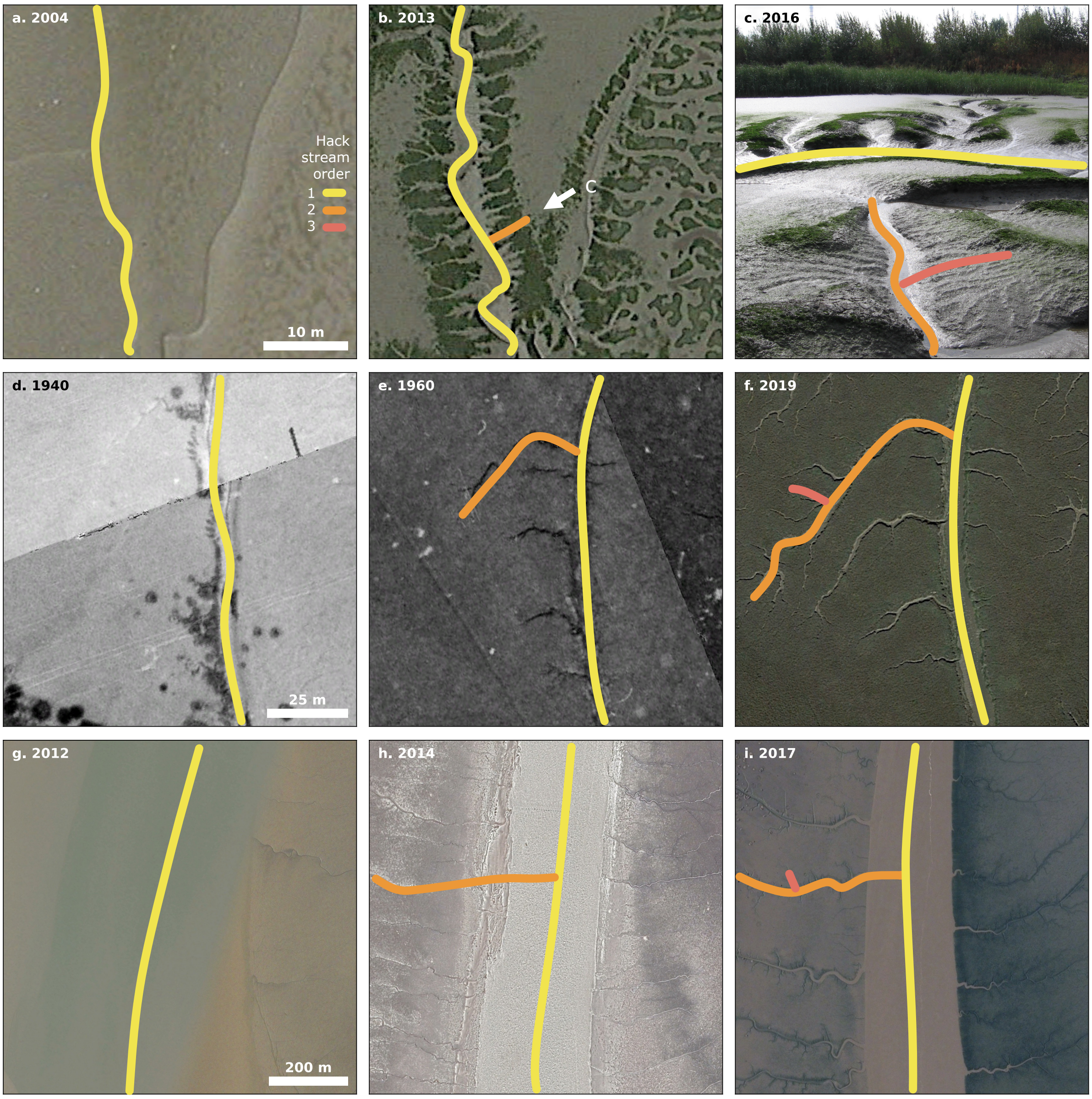

Supplementary Fig. 9 | Observed refining of tidal channel patterns under the influence of vegetation. Colored lines indicate the approximate location of Hack's 1st, 2nd and 3rd-order streams. **a-c**, Time series of channel development on the tidal flat of Ketenisse, Belgium (51°17'04.3"N, 4°18'45.1"E) in 2004 and 2013 (aerial images) and in 2016 (in-situ photo, taken approximately at the location and in the direction of the white arrow in figure b). Colonization of channel banks by algal mats (Vaucheria sp.) is visible as green patches. **d-f**, Channel network development in a tidal marsh near Leysdown-on-Sea, UK (51°21'52.54"N, 0°51'38.55"E). A similar sequence of increasingly fine-scale channel formation can be seen. **g-i**, Incision of increasingly fine-scaled side channels near Chongming Island, China (31°37'54.91"N, 121°46'47.17"E). All aerial images adapted from Google Earth Pro, © 2022 Google LLC. Specific copyrights: a,b: © 2022 Aerodata International Surveys; d,e: © 2022 Kent County Council; g-i: © 2022 Maxar Technologies.
